# Population-based analysis of ocular *Chlamydia trachomatis* in trachoma-endemic West African communities identifies genomic markers of disease severity

**DOI:** 10.1186/s13073-018-0521-x

**Published:** 2018-02-26

**Authors:** A. R. Last, H. Pickering, C. h. Roberts, F. Coll, J. Phelan, S. E. Burr, E. Cassama, M. Nabicassa, H. M. B. Seth-Smith, J. Hadfield, L. T. Cutcliffe, I. N. Clarke, D. C. W. Mabey, R. L. Bailey, T. G. Clark, N. R. Thomson, M. J. Holland

**Affiliations:** 1 Clinical Research Department, London School of Hygiene and Tropical Medicine, Keppel Street, London, UK; 2 Department of Pathogen Molecular Biology, London School of Hygiene and Tropical Medicine, Keppel Street, London, UK; 3 Disease Control and Elimination Theme, Medical Research Council Unit The Gambia, Fajara, Gambia; 4 Programa Nacional de Saúde de Visão, Ministério de Saúde Publica, Bissau, Guinea-Bissau; 5 Pathogen Genomics, Wellcome Trust Sanger Institute, Wellcome Trust Genome Campus, Hinxton, UK; 6 Clinical Microbiology, Universitätsspital Basel, Basel, Switzerland; 7 Applied Microbiology Research, Department of Biomedicine, University of Basel, Basel, Switzerland; 8 Molecular Microbiology Group, University of Southampton Medical School, Southampton, UK; 9 Department of Infectious Diseases Epidemiology, London School of Hygiene and Tropical Medicine, Keppel Street, London, UK

**Keywords:** *Chlamydia trachomatis*, Trachoma, Disease severity, Genome-wide association analysis, Single nucleotide polymorphisms, Pathogen genomic diversity

## Abstract

**Background:**

*Chlamydia trachomatis* (*Ct*) is the most common infectious cause of blindness and bacterial sexually transmitted infection worldwide. *Ct* strain-specific differences in clinical trachoma suggest that genetic polymorphisms in *Ct* may contribute to the observed variability in severity of clinical disease.

**Methods:**

Using *Ct* whole genome sequences obtained directly from conjunctival swabs, we studied *Ct* genomic diversity and associations between *Ct* genetic polymorphisms with ocular localization and disease severity in a treatment-naïve trachoma-endemic population in Guinea-Bissau, West Africa.

**Results:**

All *Ct* sequences fall within the T2 ocular clade phylogenetically. This is consistent with the presence of the characteristic deletion in *trpA* resulting in a truncated non-functional protein and the ocular tyrosine repeat regions present in *tarP* associated with ocular tissue localization. We have identified 21 *Ct* non-synonymous single nucleotide polymorphisms (SNPs) associated with ocular localization, including SNPs within *pmpD* (odds ratio, OR = 4.07, p* = 0.001) and *tarP* (OR = 0.34, p* = 0.009). Eight synonymous SNPs associated with disease severity were found in *yjfH* (*rlmB*) (OR = 0.13, p* = 0.037), *CTA0273* (OR = 0.12, p* = 0.027), *trmD* (OR = 0.12, p* = 0.032), *CTA0744* (OR = 0.12, p* = 0.041), *glgA* (OR = 0.10, p* = 0.026), *alaS* (OR = 0.10, p* = 0.032), *pmpE* (OR = 0.08, p* = 0.001) and the intergenic region *CTA0744–CTA0745* (OR = 0.13, p* = 0.043).

**Conclusions:**

This study demonstrates the extent of genomic diversity within a naturally circulating population of ocular *Ct* and is the first to describe novel genomic associations with disease severity. These findings direct investigation of host-pathogen interactions that may be important in ocular *Ct* pathogenesis and disease transmission.

**Electronic supplementary material:**

The online version of this article (10.1186/s13073-018-0521-x) contains supplementary material, which is available to authorized users.

## Background

The obligate intracellular bacterium *Chlamydia trachomatis (Ct)* is the leading infectious cause of blindness (trachoma) and the most common sexually transmitted bacterial infection [1, 2].

*Ct* strains are differentiated into biovars based on pathobiological characteristics and serovars based on serological reactivity for the major outer membrane protein (MOMP) encoded by *ompA* [3]. Serovars largely differentiate biological groups associated with trachoma (A–C), sexually transmitted disease (D–K) and lymphogranuloma venereum (LGV) (L1–L3). Despite diverse biological phenotypes, *Ct* strains share near complete genomic synteny and gene content [4], suggesting that minor genetic changes influence pathogen-host and tissue-specific infection characteristics [5–8]. All published African ocular *Ct* genomes are situated on the ocular branch within the T2 clade of non-LGV urogenital isolates [4]. Currently there are only 31 published ocular *Ct* genome sequences [4, 9–12].

The pathogenesis of chlamydial infection begins with epithelial inflammation and may progress to chronic immunofibrogenic processes leading to blindness and infertility, though many *Ct* infections do not result in sequelae [13, 14]. Strain-specific differences related to clinical presentation have been investigated in trachoma [8, 15, 16]. These studies examined a small number of ocular *Ct* isolates from the major trachoma serotypes and found a small subset of genes in addition to *ompA* that were associated with differences in in vitro growth rate, burst size, plaque morphology, interferon gamma –(IFNγ) sensitivity and, most importantly, intensity of infection and clinical disease severity in non-human primates (NHPs), suggesting that genetic polymorphisms in *Ct* may contribute to the observed variability in severity of trachoma in endemic communities [8].

The obligate intracellular development of *Ct* has presented significant technical barriers to basic research into chlamydial biology. Only recently has genetic manipulation of the chlamydial plasmid been possible, allowing in vitro transformation and modification studies, though this remains technically challenging, necessitating alternative approaches [17, 18].

Whole genome sequencing (WGS) has recently been used to identify regions of likely recombination in recent clinical isolates, demonstrating that WGS analysis may be an effective approach for the discovery of loci associated with clinical presentation [6]. Additionally, a number of putative virulence factors have been identified through WGS analysis and subsequent in vitro and animal studies [5, 19–30]. However, there are currently no published population-based studies of *Ct* using WGS with corresponding detailed clinical data, making it difficult to relate genetic changes to functional relevance and virulence factors in vivo.

There is an increasing pool of *Ct* genomic data, largely from archived samples following cell culture and more recently directly from clinical samples [31]. WGS data obtained directly from clinical samples can be preferable to using WGS data obtained from cell-cultured *Ct*, since repeated passage of *Ct* results in mutations that are not observed in vivo [32–34].

*Ct* bacterial load is associated with disease severity, particularly conjunctival inflammation, in active (infective) trachoma [35]. Conjunctival inflammation has previously been shown to be a marker of severe disease and plays an important role in the pathogenesis of scarring trachoma [36–38]. In this study we used principal component analysis (PCA) to reduce the dimensions of clinical grade of inflammation (defined using the P score from the follicles, papillary hypertrophy, conjunctival scarring (FPC) trachoma grading system [39]) and *Ct* bacterial load to a single metric to define an in vivo conjunctival phenotype in active (infective) trachoma. PCA is a recognized dimension reduction technique used to combine multiple correlated traits into their uncorrelated principal components (PCs) [40–42], allowing us to examine the relationship between *Ct* genotype and disease severity. These data from the trachoma-endemic region of the Bijagós Archipelago of Guinea-Bissau currently represent the largest collection of ocular *Ct* sequences from a single population and provide a unique opportunity to gain insight into ocular *Ct* pathogenesis in humans.

## Methods

### Survey, clinical examination and sample collection

Survey, clinical examination and sample collection methods have been described previously [43, 44]. Briefly, we conducted a cross-sectional population-based survey in trachoma-endemic communities on the Bijagós Archipelago of Guinea-Bissau. The upper tarsal conjunctivae of each consenting participant were examined, digital photographs were taken, a clinical trachoma grade was assigned and two sequential conjunctival swabs were obtained from the left upper tarsal conjunctiva of each individual using a standardized method [43]. DNA was extracted and *Ct omcB* (genomic) copies/swab quantified from the second conjunctival swab using droplet digital polymerase chain reaction (ddPCR) [44, 45].

We used the modified FPC grading system for trachoma [39]. The modified FPC system allows detailed scoring of the conjunctiva for the presence of follicles (F score), papillary hypertrophy (conjunctival inflammation) (P score) and conjunctival scarring (C score), assigning a grade of 0–3 for each parameter. A single validated grader conducted the examinations, and these were verified by an expert grader (masked to the field grades and ddPCR results) using the digital photographs. Grader concordance was measured using Cohen’s kappa, where a kappa > 0.9 was used as the threshold to indicate good agreement.

**Fig. 1:**
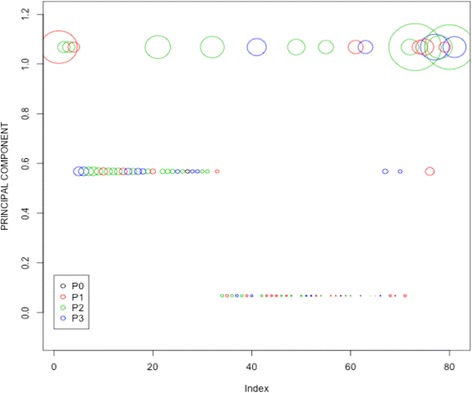
Composite in vivo conjunctival disease severity phenotype in ocular *Chlamydia trachomatis* infection. A composite in vivo phenotype was derived using principal component analysis (PCA) for dimension reduction of two phenotypic traits: a disease severity score (using the P score value) and *C. trachomatis* load (where *C. trachomatis* load was log transformed and cut-offs determined from the resulting density plot (see Additional file 1: Figure S1)). Each *circle* represents an individual infection (represented on the *x*-axis (Index), *n* = 81). *Circle size* reflects *C. trachomatis* load and *circle colour* reflects inflammatory P score (P0–P3) defined using the modified FPC (follicles, papillary hypertrophy, conjunctival scarring) grading system for trachoma [39]

Conjunctival inflammation (P score) is known to have a strong association with *Ct* bacterial load in this and other populations [35, 46–49]. For this study we used PCA to combine the presence of inflammation (defined by the P score using the FPC trachoma grading system [39]) with *Ct* bacterial load (defined by tertile cut-offs illustrated in Additional file 1: Figure S1) [50]. The conjunctival disease phenotype is a dimension reduction of these two variables, defining what we observed in the conjunctiva at the time of sampling (Fig. 1). Dimension reduction using PCA to define complex disease phenotypes in genome-wide association studies (GWASs) is well recognized, as it allows multiple traits to be included to capture a more complex phenotype and accounts for correlation between traits. This approach therefore may reveal novel loci or pathways that would not be evident in a single-trait GWAS, where the full extent of genetic variation cannot be captured [40].

### Preparation of chlamydial DNA from cell culture

For eight specimens, WGS data were obtained following *Ct* isolation in cell culture (from the first conjunctival swab) as a preliminary exploration of *Ct* genomic diversity in this population. Briefly, samples were isolated in McCoy cell cultures by removing 100 μl eluate from the original swab with direct inoculation onto a glass coverslip within a bijou containing Dulbecco’s modified Eagle’s medium (DMEM). The inocula were centrifuged onto cell cultures at 1800 rpm for 30 min. Following centrifugation the cell culture supernatant was removed and cycloheximide-containing DMEM was added to infected cells which were then incubated at 37 °C in 5% CO_2_ for 3 days. Viable *Ct* elementary bodies (EBs) were observed by phase contrast microscopy. Cells were harvested and further passaged every 3 days until all isolates reached a multiplicity of infection between 50 and 90% in 2xT25 flasks. Each isolate was prepared and the EBs purified as described previously [51]. DNA was extracted from the purified EBs using the Promega Wizard Genomic Purification kit according to the manufacturer’s protocol [52].

### Pre-sequencing target enrichment

For the remaining specimens (*n =* 118), WGS data were obtained directly from clinical samples. DNA baits spanning the length of the *Ct* genome were compiled by SureDesign and synthesized by SureSelect^XT^ (Agilent Technologies, UK). The total DNA extracted from clinical samples was quantified and carrier human genomic DNA added to obtain a total of 3 μg input for library preparation. DNA was sheared using a Covaris E210 acoustic focusing unit [31]. End-repair, non-templated addition of 3′-A adapter ligation, hybridization, enrichment PCR and all post- reaction clean-up steps were performed according to the SureSelect^XT^ Illumina Paired-End Sequencing Library protocol (v1.4.1 Sept 2012). All recommended quality control measures were performed between steps.

### Whole genome sequencing and sequence quality filtering

DNA was sequenced at the Wellcome Trust Sanger Institute using Illumina paired-end technology (Illumina GAII or HiSeq 2000). All 126 sequences passed standard FastQC quality control criteria [53]. Sequences were aligned to the most closely related reference genome, *Chlamydia trachomatis A/HAR-13* (GenBank accession umber NC_007429.1 and plasmid GenBank accession number NC_007430.1), using the Burrows-Wheeler Aligner (BWA) [54]. SAMtools/BCFtools (SAMtools v1.3.1) [55] and the Genome Analysis Tool Kit (GATK) [56] were used to call SNPs. We used standard GATK SNP calling algorithms, where > 10× depth of coverage is routinely used as the threshold value [56, 57]. This has been shown to be adequate for SNP calling in this context [57–59].

Variants were selected as the intersection data set between those obtained using both SNP callers and SNPs were further quality-filtered. SNP alleles were called using an alternative coverage-based approach where a missing call was assigned to a site if the total coverage was less than 20× depth or where one of the four nucleotides accounted for at least 80% total coverage [60]. There was a clear relationship between the mean depth of coverage and the proportion of missing calls, based on which we retained sequences with greater than 10× mean depth of coverage over the whole genome (81 sequences retained).

**Fig. 2:**
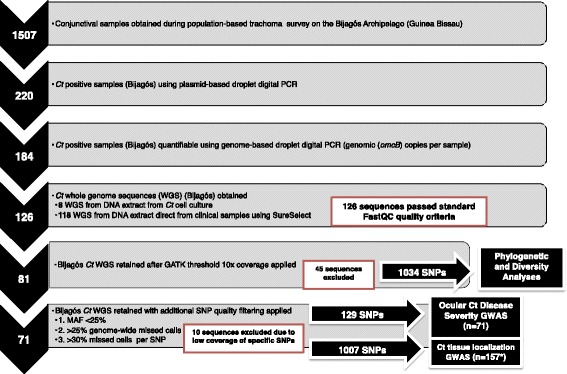
Whole genome sequencing (WGS) quality filtering processes and threshold criteria for inclusion in analyses. *Ct* DNA detected using droplet digital PCR [45]. WGS data were obtained using SureSelect target enrichment [31] (or chlamydial cell culture) and Illumina paired-end sequencing. FastQC [53] was used to assess basic WGS quality. SNP alleles were called against reference strain *Ct A/HAR-13* using an alternative coverage-based approach where a missing call was assigned to a site if the total coverage was less than 20× depth or where one of the four nucleotides accounted for at least 80% total coverage [60]. There was a clear relationship between the mean depth of coverage and genome-wide proportion of missing calls; therefore, only sequences with greater than 10× mean depth of coverage over the whole genome were retained using the GATK Best Practices threshold [56, 57]. Heterozygous calls were removed and SNPs with a minor allele frequency (MAF) of less than 25% were removed. Samples with greater than 25% genome-wide missing data and 30% missing data per SNP were excluded from the analysis. WGS sequence quality is shown in detail in Additional file 12: Figure S12. *n = 157 including the 71 Bijagós sequences in addition to 48 Rombo District sequences and 38 reference sequences

Heterozygous calls were removed, and SNPs with a minor allele frequency (MAF) of less than 25% were removed. Samples with greater than 25% genome-wide missing data and 30% missing data per SNP were excluded from the analysis (*n* = 10, 71 sequences retained). All SNP positions with a MAF greater than 20% were identified using BCFtools v0.1.19 (https://samtools.github.io/bcftools/). Sequences were excluded from the final GWAS if more than 300 such positions were found using methods described by Hadfield et al. [61]. The quality assessment and filtering process is shown in Fig. 2. Details of the WGS data are provided in Additional file 2: Figure S2.

### Phylogenetic reconstruction

Samples were mapped to the ocular reference strain *Ct A/HAR-13* and SNPs were called as described above. Phylogenies were computed using RAxML v7.8.2 [62] from a variable sites alignment using a generalized time-reversible (GTR) + gamma model and are midpoint rooted. Recombination is known to occur in *Ct* [4, 6] and can be problematic in constructing phylogeny. We applied three compatibility-based recombination detection methods to detect regions of recombination using PhiPack [63]: the pairwise homoplasy index (Phi), the maximum χ^2^ and the neighbour similarity score (NSS) across the genome alignment. We also examined the confidence in the phylogenetic tree by computing RAxML site-based likelihood scores [62]. Phylogenetic trees were examined adjusting for recombination using the methods described above.

Additionally, sequence data for the tryptophan operon (*CTA0182* and *CTA0184*–*CTA0186*), *tarP* (*CTA0498*), nine polymorphic membrane proteins (*CTA0447*–*CTA0449, CTA0884, CTA0949*–*CTA0952* and *CTA0954*) and *ompA* (*CTA0742*) were extracted from the 81 ocular *Ct* sequences from Guinea-Bissau retained after quality control filtering described above, 48 ocular sequences originating from a study conducted in Kahe village, Rombo District, Tanzania [64] and 38 publicly available reference sequences. Phylogenies were constructed as described above.

Polymorphisms, insertions and deletions (indels) and truncations for the tryptophan operon were manually determined from aligned sequences using SeaView [65]. Tyrosine repeat regions and actin-binding domains in *tarP* were found using RADAR [66] and Pfam [67] respectively.

### Pairwise diversity

A comparison was made between the two population-based *Ct* sequence data sets from the Bijagós (Guinea-Bissau) and Rombo (Tanzania) sequences whereby short read data from the 81 Bijagós sequences and 48 Rombo sequences were mapped against *Ct A/HAR-13* using SAMtools. Within-population pairwise nucleotide diversity was calculated using the formula:

$$ \pi =2\times {\varSigma}_{i=1}^n\ {\varSigma}_{j=1}^{i-1}{x}_i{x}_j{\pi}_{ij} $$

where *n* is the number of sequences, *x* is the frequency of sequences *i* and *j* and π_*ij*_ is the number of nucleotide differences per site between sequences *i* and *j* [68]. The frequency of sequences was considered uniform within the populations, and sites with missing calls were excluded on a per-sequence basis.

### Genome-wide association analyses

To investigate the association between *Ct* polymorphisms with ocular localization and clinical disease severity, we used permutation-based logistic regression methods, which are powerful and well-recognized tools in GWAS, allowing for adjustment for population structure, age and gender in the model and accounting for multiple testing [69–72].

We used permutation analyses of 100,024 phenotypic re-samplings, where the distribution of the *p* value was approximated by simulating data sets through randomization under the null hypothesis of no association between phenotype and genotype. Genome-wide significance was determined as p* ≤ 0.05, where p* was defined as the fraction of re-sampled (simulated) data that returned *p* values that were less than or equal to the *p* values observed in the data [50]. All analyses were conducted using the R statistical package v3.0.2 (the R Foundation for Statistical Computing, https://www.r-project.org/) using MASS, GLM and lsr. All R script used for these analyses is contained within Additional file 3: Figure S3 and is released as a CC-BY open resource (CC-BY-SA 3.0).

### Ocular localization

Tissue localization is defined as the localization (or presence) of a detectable *Ct* infection to either the conjunctival epithelium or the urogenital tract. Short read data from the 129 clinical ocular sequences from the pairwise diversity analysis and 38 publicly available reference sequences from ocular (*n* = 8), urogenital (*n* = 17) and rectal (*n* = 13) sites were mapped against *Ct A/HAR-13* using SAMtools. Only polymorphic sites were retained, and SNPs were filtered as described above. The final analysis includes 1007 SNPs from 157 sequences, a phylogeny of which is contained within Additional file 4: Figure S4. A permutation-based generalized linear regression model was used to test the association between collection site (ocular or urogenital tissue localization) and polymorphic sites. For each SNP the standard error for the *t* statistic was estimated from the model and used to calculate the odds ratios (ORs) and 95% confidence intervals. A χ^2^ test was used to determine the association between ocular localization-associated SNPs and both gene expression stage and predicted localization of the encoded proteins. The developmental cycle expression stage for each transcript was based on data and groupings from Belland et al. [73]. Predicted localization of expressed proteins was defined using the consensus from three predictions using CELLO [74], PSORTb [75] and LocTree3 [76].

### Clinical disease severity

A permutation-based ordinal logistic regression model was used to test the association between the disease severity score (using the in vivo conjunctival phenotype defined previously) and polymorphic sites. The final analysis includes 129 SNPs from 71 sequences derived as described in Fig. 2. For each SNP the standard error for the *t* statistic was estimated from the model and used to calculate the ORs and 95% confidence intervals. Individuals’ age and gender were included as a covariate to the regression analysis.

We investigated the effect of population structure on the results of the GWAS analysis using PCA [77]. The first three PCs captured the majority of structural variation, but including them in the model had no effect; therefore, they were not included in the final model.

We corrected for genomic inflation if the occurrence of a polymorphism in the population was more than 90% or if there was a MAF of 3%.

## Results

Conjunctival swabs collected during a cross-sectional population-based trachoma survey on the Bijagós Archipelago yielded 220 ocular *Ct* infections detected by *Ct* plasmid-based ddPCR. Of the 220 *Ct* infections detected, 184 were quantifiable using *Ct* genome-based ddPCR.

We obtained WGS data from 126/220 samples using cell culture (*n* = 8) or direct sequencing from swabs with SureSelect^XT^ target enrichment (*n* = 118), representing the largest cross-sectional collection of ocular *Ct* WGS. Eighty-one of these sequences were subsequently included in the phylogenetic and diversity analyses and 71 were retained in the final genome-wide association (tissue localization (derived from the anatomical site of sample collection) and disease severity) analyses. The quality filtering process is illustrated in Fig. 2 and detailed in Methods.

A total of 1034 unique SNP sites were identified within the 126 Bijagós *Ct* genomes relative to the reference strain *Ct A/HAR-13*. Following application of further threshold criteria based on MAF and genome-wide missing data thresholds, we retained only high-quality genomic data in the final association analyses (129 SNPs from 71 sequences). There were no significant differences between the 71 retained and the 55 excluded sequences with respect to demographic characteristics, bacterial load, disease severity scores or geographical location (Table 1). Clinical and demographic details of the survey participants in whom we did not identify *Ct* infection have been published previously [43]. Of the ten SNPs initially identified within the *Ct* plasmid sequences, none fulfilled the quality filtering criteria, and they were not retained for the genome-wide association analyses.

**Table 1:** Characteristics of ocular *Chlamydia trachomatis* sequences included in the disease severity association analysis

Sequence ID	Sample ID	Average depth of coverage	% Missing reads^a^	Gender	Age (years)	Island code	Village code	Ocular load^b^	P score^c^
11152_3_1	14,344	764	0.35%	M	4	002	33	202,632	1
11152_3_10	17,347	121	0.21%	M	5	001	17	69,093	2
11152_3_11	4422	19	19.95%	F	2	001	12	68,782	2
11152_3_12	11,231	68	2.24%	M	0	003	43	64,036	1
11152_3_13	15,631	21	14.93%	F	2	002	33	55,749	3
11152_3_14	6105	1664	0.05%	F	1	001	14	55,202	3
11152_3_15	12,628	191	0.10%	F	12	002	29	54,651	2
11152_3_16	7524	2065	0.14%	M	10	002	35	54,539	2
11152_3_17	5016	61	0.44%	F	1	001	15	46,510	2
11152_3_18	1485	44	1.21%	F	4	002	27	45,929	1
11152_3_19	15,554	825	0.06%	F	1	002	33	44,052	2
11152_3_20	6094	3070	0.00%	F	3	001	14	42,917	2
11152_3_22	5082	51	0.81%	M	6	001	15	42,427	1
11152_3_23	12,969	3643	1.81%	F	3	002	29	41,308	3
11152_3_25	8140	246	0.36%	M	13	001	20	39,816	2
11152_3_26	6083	2746	0.00%	F	23	001	14	38,771	3
11152_3_27	16,621	1664	0.00%	M	3	002	37	33,514	3
11152_3_28	16,852	143	0.16%	M	5	002	38	31,228	2
11152_3_29	16,588	53	0.81%	M	6	002	37	29,991	1
11152_3_3	4180	51	0.92%	M	2	001	12	140,693	2
11152_3_30	7612	107	0.44%	F	3	002	35	28,528	2
11152_3_31	6985	177	0.10%	M	6	001	17	27,924	2
11152_3_32	4411	24	9.68%	F	1	001	12	27,584	2
11152_3_33	4257	381	0.06%	M	0	001	12	24,033	3
11152_3_34	4400	48	0.98%	M	6	001	12	23,435	2
11152_3_35	15,180	571	0.35%	F	7	002	33	23,254	0
11152_3_36	13,596	496	0.06%	M	18	002	23	22,098	3
11152_3_37	1672	20	18.42%	M	6	002	25	21,630	3
11152_3_38	5181	81	0.32%	M	4	001	15	21,339	2
11152_3_39	15,532	243	0.08%	F	25	002	33	21,174	2
11152_3_4	8074	150	0.13%	M	4	001	18	131,175	2
11152_3_40	16,984	145	0.19%	M	4	002	21	20,113	1
11152_3_41	1881	37	2.71%	F	1	002	32	15,963	2
11152_3_42	10,032	101	0.16%	M	2	003	42	15,706	1
11152_3_43	8492	70	2.60%	M	1	004	45	15,582	2
11152_3_44	13,585	31	4.97%	M	23	002	23	15,417	3
11152_3_48	7535	61	0.84%	M	18	002	35	13,439	3
11152_3_5	7095	235	0.44%	F	4	001	17	105,453	3
11152_3_50	6028	46	1.24%	F	4	001	14	12,961	2
11152_3_52	10,021	20	16.15%	F	6	003	42	11,840	1
11152_3_55	12,650	59	0.54%	M	6	002	29	9001	2
11152_3_57	8965	21	16.60%	M	27	003	43	7336	1
11152_3_58	5104	33	3.68%	M	2	001	15	7203	2
11152_3_6	16,599	52	0.73%	M	9	002	37	96,333	2
11152_3_62	7062	22	13.41%	F	4	001	17	6986	3
11152_3_63	8778	17	25.47%	F	11	004	46	6760	3
11152_3_66	1892	45	1.25%	F	2	002	32	6374	1
11152_3_7	10,747	581	1.82%	F	3	003	44	82,916	2
11152_3_70	13,189	25	8.87%	F	3	002	24	4703	1
11152_3_74	15,499	24	10.49%	M	5	002	33	4226	1
11152_3_76	726	417	0.06%	F	3	002	26	3753	0
11152_3_77	7579	105	0.52%	F	5	002	35	3468	1
11152_3_78	12,089	16	27.78%	F	13	002	47	3203	2
11152_3_8	6996	38	2.03%	M	3	001	17	82,614	1
11152_3_88	748	163	0.10%	F	2	002	26	1636	0
11152_3_9	10,967	20	17.52%	F	2	003	44	81,124	3
11152_3_92	1463	73	0.30%	F	42	002	27	1273	2
13108_1_14	24,519	51	2.81%	M	2	004	45	29,040	3
13108_1_15	6941	33	1.81%	M	36	001	17	13,155	1
13108_1_7	25,124	27	5.27%	M	4	002	22	21,750	3
13108_1_9	22,154	18	20.56%	F	5	003	43	14,349	1
8422_8_49	2353	39	5.70%	M	11	002	35	96,889	2
8422_8_50	2366	82	1.08%	M	1	002	35	289,778	2
9471_4_86	12,980	287	1.90%	M	4	002	29	85,456	1
9471_4_87	15,367	215	0.46%	M	1	002	33	99,064	1
9471_4_88	15,543	192	0.11%	F	23	002	33	49,125	1
9471_4_89	1870	119	0.14%	M	3	002	32	158,548	3
9471_4_90	2145	111	0.11%	M	15	002	32	140,297	2
9471_4_91	4158	94	0.14%	M	4	001	12	63,654	1
9471_4_92	4169	85	0.13%	F	3	001	12	274,835	2
9471_4_93	7590	242	0.51%	F	1	002	35	128,025	3

Sequences (*n* = 55) were excluded from the association analysis if there was (1) < 10× coverage, (2)^a^ > 25% missing reads genome-wide and (3) > 25% missing (*N*) calls at the single nucleotide polymorphism (SNP) locus. Coverage and missing data were correlated and resulted in exclusion of the same samples irrespective of criteria chosen. Seventy-one sequences were retained in the final disease severity analysis. ^b^Ocular *C. trachomatis* load = *omcB* (*C. trachomatis* genome) copies per conjunctival swab measured using droplet digital PCR. ^c^P score = conjunctival inflammation score (0–3) using the modified FPC (follicles, papillary hypertrophy, conjunctival scarring) grading system for trachoma [39]

### Ocular *C. trachomatis* phylogeny and diversity

**Fig. 3:**
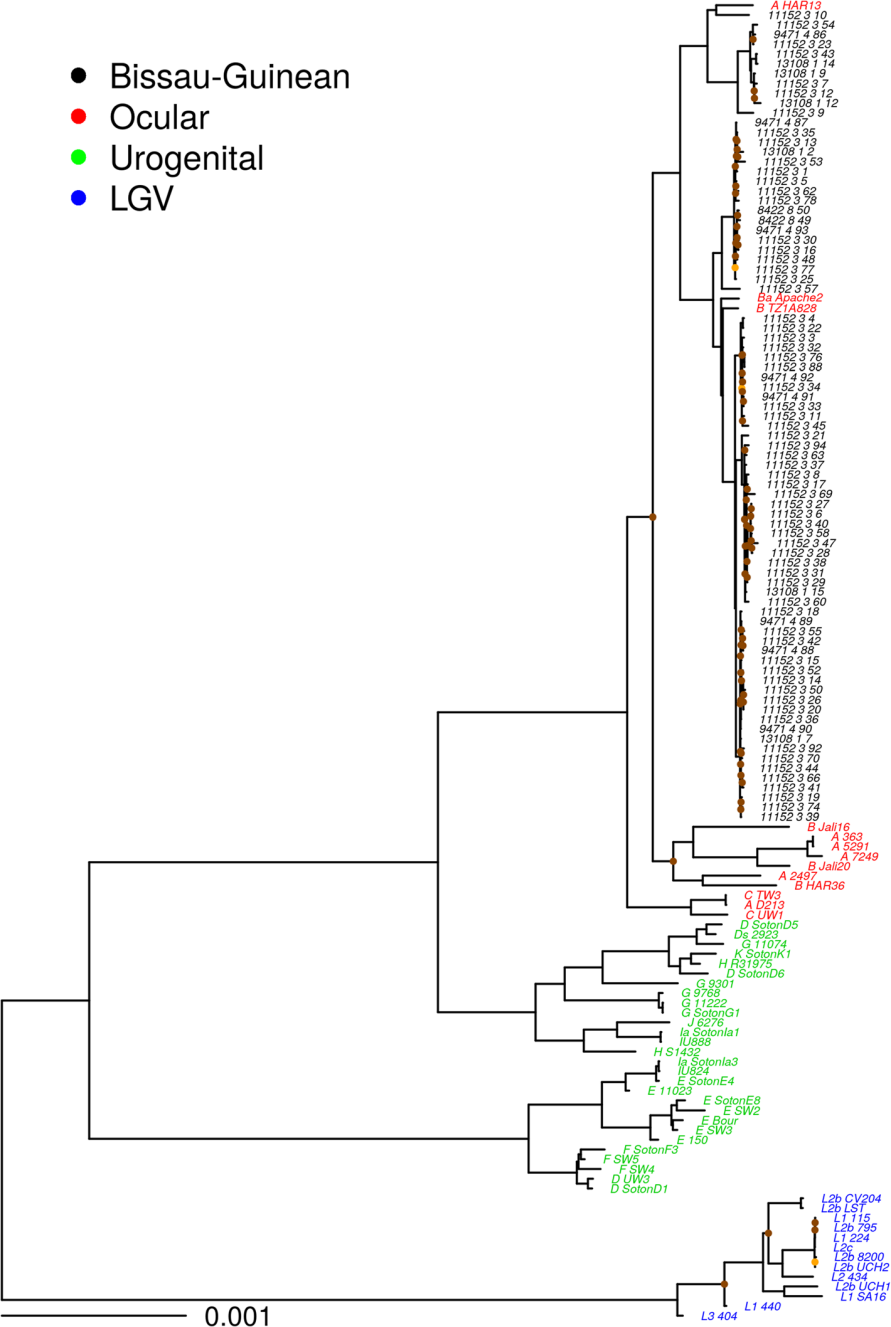
Maximum likelihood reconstruction of whole genome phylogeny of ocular *Chlamydia trachomatis* sequences from the Bijagós Archipelago (Guinea-Bissau). Maximum likelihood reconstruction of the whole genome phylogeny of 81 *Ct* sequences from the Bijagós Islands and 54 *Ct* reference strains. Bijagós *Ct* sequences (*n* = 81) were mapped to *Ct A/HAR-13* using SAMtools [55]. SNPs were called as described by Harris et al. [4]. Phylogenies were computed with RAxML [62] from a variable sites alignment using a GTR + gamma model and are midpoint rooted. The *scale bar* indicates evolutionary distance. Bijagós *Ct* sequences in this study are coloured *black*, and reference strains are coloured by tissue localization (*red* = Ocular, *green* = Urogenital, *blue* = LGV). Branches are supported by > 90% of 1000 bootstrap replicates. Branches supported by 80–90% (*orange*) and < 80% (*brown*) bootstrap replicates are indicated

For the phylogeny and diversity analyses, 81 Bijagós *Ct* sequences were included on the basis of the quality filtering criteria described in detail in Fig. 2. SNP-based phylogenetic trees constructed using all 1034 SNPs for sequences above 10× coverage (*n* = 81), with 54 published *Ct* reference genomes, are shown in Fig. 3.

The Bijagós sequences are situated within the T2 ocular monophyletic lineage with all other ocular *Ct* sequences [59] except those described by Andersson et al. [10]. However, our population-based collection of ocular *Ct* sequences has much greater diversity at whole genome resolution than previously demonstrated in African trachoma isolates [4, 8]. We used a pairwise diversity (π) metric to compare two populations of ocular *Ct* from regions with similar trachoma endemicity and studies with similar design, sample size and available epidemiological metadata. These data show much greater genomic diversity in the Bijagós ocular *Ct* sequences (π = 0.07167) compared to the Tanzanian (Rombo) ocular *Ct* sequences (π = 0.00047).

**Fig. 4:**
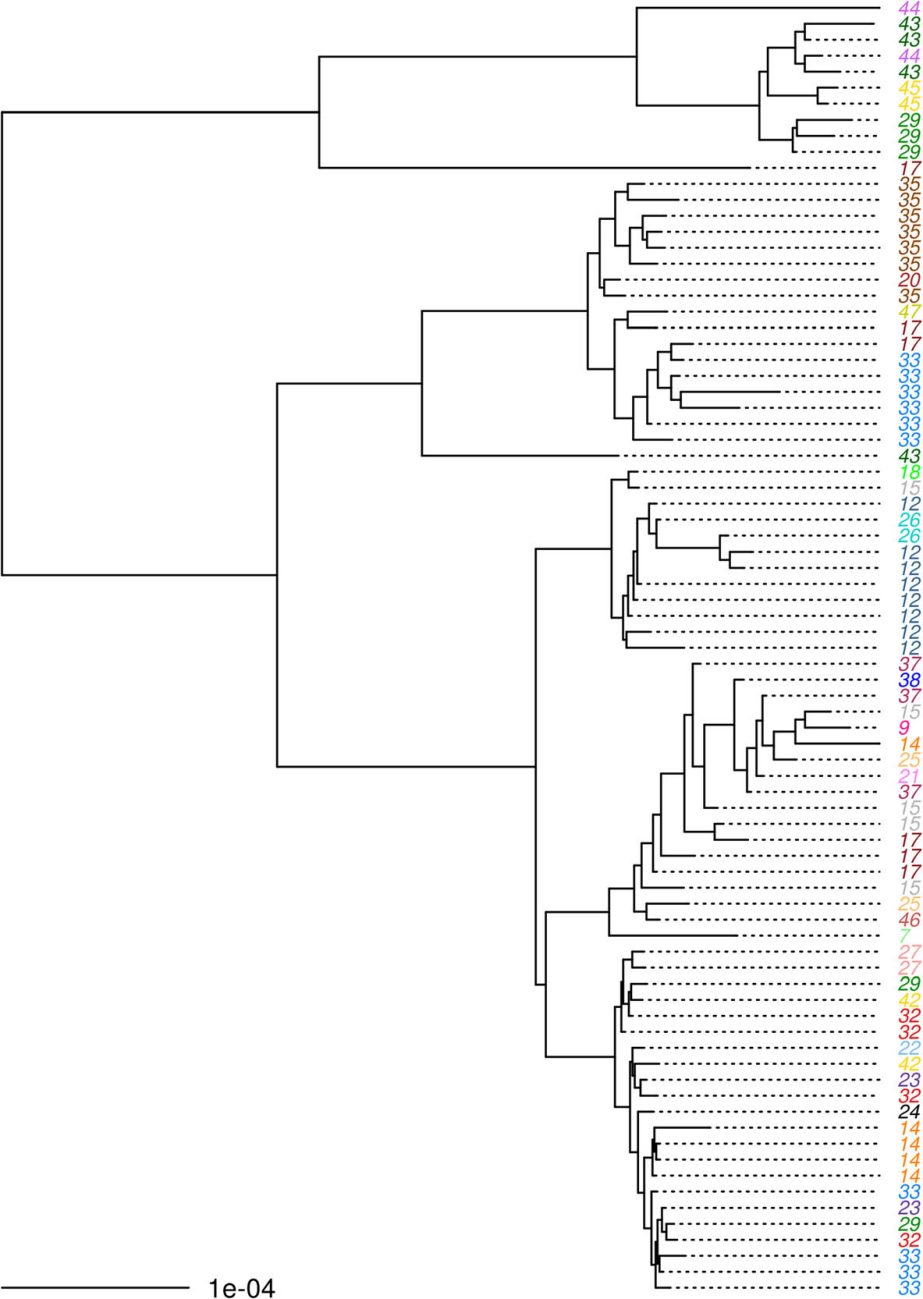
Maximum likelihood phylogenetic tree showing clustering of ocular *Chlamydia trachomatis* sequence types by village. RAxML maximum likelihood phylogenetic reconstruction including all ocular *Ct* sequences retained in the final disease severity association analysis after quality filtering (*n* = 71). Ocular *Ct* sequences are labelled by village (villages numbered and coloured), midpoint-rooted and mapped to reference *Ct A/HAR-13*. Branches are supported by > 90% of 1000 bootstrap replicates. Branches supported by 80–90% (*orange*) and < 80% (*brown*) bootstrap replicates are indicated

By *ompA* genotyping, 73 of the Bijagós sequences are genotype A and 8 are genotype B, supporting their classical ocular nature (Additional file 5: Figure S5). The high resolution of WGS data obtained directly from clinical samples captures diversity that may be useful in strain classification, particularly as we found some evidence of clustering at village level, although the very small number of sequences per village means that it is not possible to provide accurate estimates of clustering in this study (Fig. 4).

Homoplasic SNPs and regions affected by recombination are shown in Additional file 6: Figure S6a. Removal of these regions of recombination identified using the pairwise homoplasy index had no effect on phylogenetic relationships. Additionally, a site-wise log likelihood plot demonstrated that there was no clear genomic region where there was significant lack of confidence in the tree construction due to recombination (Additional file 6: Figure S6b). Whether regions containing recombination were included or excluded, tree topology remained essentially identical, indicating that branching order is not affected by the removal of these regions.

### Genome-wide analysis of *C. trachomatis* localization

Candidate genes thought to be involved in or indicative of ocular localization or preference were examined to further characterize this population of ocular *Ct*. Polymorphisms and truncations in the tryptophan operon have previously been implicated in the inability of ocular *Ct* to infect and survive in the genital tract [5]. All sequences contained mutations in *trpA* resulting in truncation. The majority (80/81) were truncated at the previously characterized deletion at position 533 [5]. Polymorphisms in *trpB* and *trpR* were less common (Additional file 7: Figure S7).

The variable domain structure of the translocated actin-recruiting phosphoprotein (*tarP*) has also been implicated in tropism [78]. Ocular strains possess more actin-binding domains (three or four) and fewer tyrosine repeat regions (between one and three). Urogenital strain *tarP* sequences have low copy numbers of both, and LGV strain sequences have additional tyrosine repeat regions. In this study, all sequences contain the expected three tyrosine repeat regions and three or four actin-binding domains (Additional file 7: Figure S7).

The nine virulence-associated polymorphic membrane proteins (Pmp) are variably related to tissue preference, with all encoding genes except *pmpA*, *pmpD* and *pmpE* clustering by tissue location [20]. In this population all phylogenies of the six tropism-clustering *pmps* show that all sequences cluster with other ocular sequences (Additional file 8: Figure S8).

**Fig. 5:**
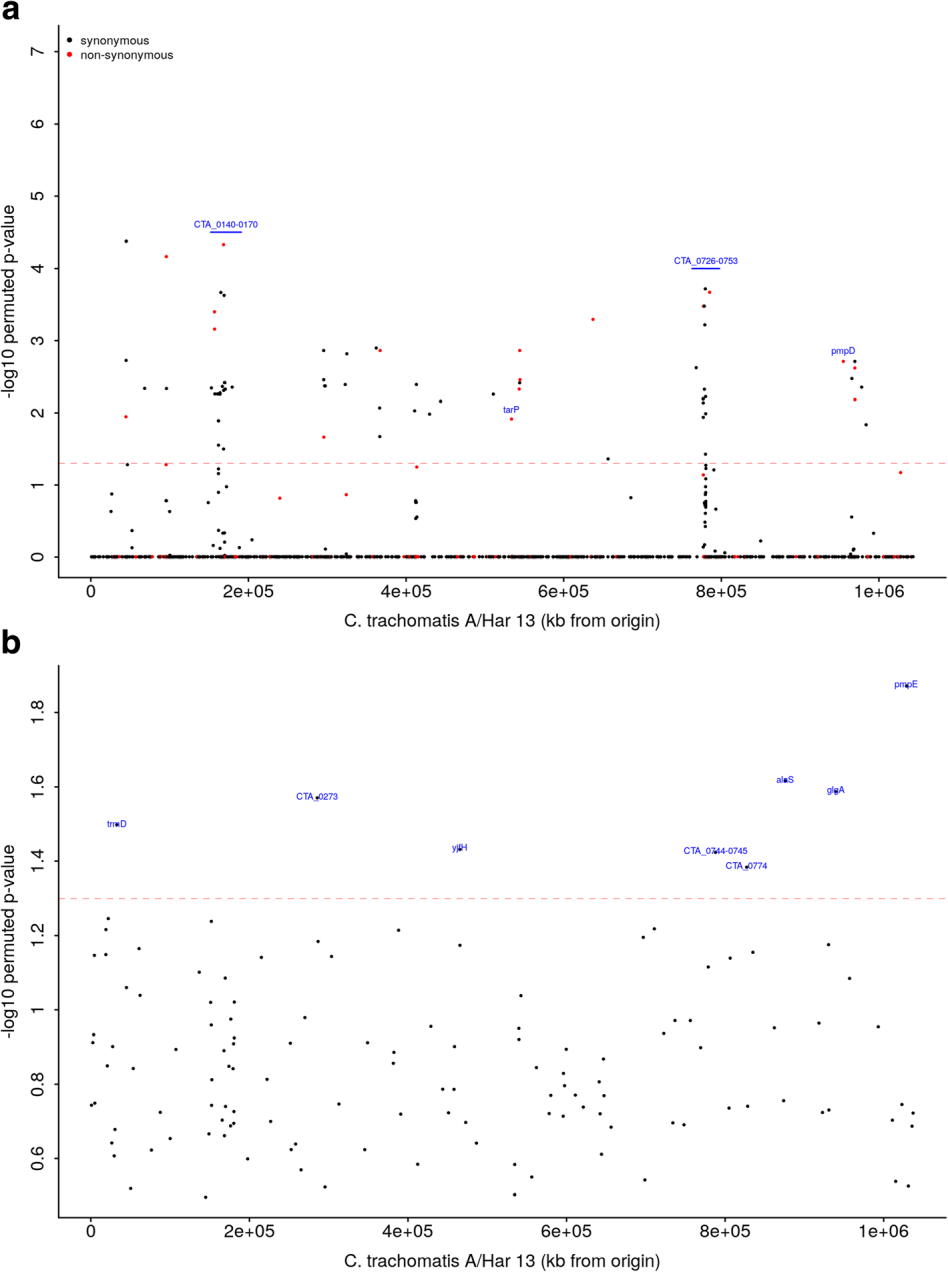
Single nucleotide polymorphisms on the *Chlamydia trachomatis* genome associated with (**a**) ocular localization and (**b**) disease severity at genome-wide significance. **a** Ocular localization-associated SNPs across the *C. trachomatis* genome. There were 1007 SNPs identified in coding and non-coding regions and included in permutation-based linear regression models in the *Ct* genome-wide association analysis. The threshold for genome-wide significance is indicated by the *dashed line* (p* < 0.05). The *y*-axis shows the –log10 *p* value. A –log10 *p* value of 1.3 is equivalent to a permuted *p* value of 0.05 (p* < 0.05). Synonymous (*black*) and non-synonymous SNPs (*red*) are indicated. Regions informative for ocular localization and genes of interest are labelled in *blue*. **b** Disease severity-associated SNPs across the *Ct* genome. From 129 SNPs identified in coding and non-coding regions, SNPs associated with the disease severity phenotype at genome-wide significance are identified using permutation-based ordinal logistic regression models adjusting for age in the *Ct* genome-wide association analysis. The threshold for genome-wide significance is indicated by the *dashed line* (p* < 0.05). The *y*-axis shows the –log10 *p* value. A log10 *p* value of 1.3 is equivalent to a permuted *p* value of 0.05 (p* < 0.05). Genes significantly associated with disease severity are labelled in *blue*

Permutation-based re-sampling methods, commonly used in GWAS analyses, were used to account for multiple comparisons [69–72]. We tested 1007 SNPs in 157 *Ct* sequences (Fig. 2) for association with ocular localization (defined by anatomical site of sample collection), comparing 127 ocular, 17 urogenital and 13 LGV strains (Fig. 5a). One hundred and five SNPs were significantly associated with ocular localization (p* < 0.05), of which 21 were non-synonymous (details in Table 2a and Additional file 9: Figure S9). These were within a number of genes known to be polymorphic, genes previously identified as tropism-associated (*CTA0156*, *CTA0498*/*tarP* and *CTA0743*/*pbpB*) and virulence factors (*CTA0498*/*tarP* and *CTA0884*/*pmpD*). Four genes contained multiple non-synonymous SNPs (*CTA_0733/karG*, *CTA_089/5sucD*, *CTA_0087* and *CTA_0145/oppA_1*), and ten genes contained multiple synonymous SNPs. Of the genes containing multiple synonymous SNPs, five contained more than three SNPs (*CTA_0739/tsf*, *CTA_0733/karG*, *CTA_0156*, *CTA_0154* and *CTA_0153*). No predicted protein localization was over-represented in the ocular localization-related SNPs (*p* = 0.6174); however, early and very-late expressed genes were over-represented (*p* = 0.0197).

**Table 2:** SNPs across the *Chlamydia trachomatis* genome identified using permutation-based genome-wide association analysis for (A) ocular localization (non-synonymous only) and (B) disease severity

(A)															
SNP position	Ocular allele (%)	Urogenital allele (%)	Name *A/HAR-13*	CDS	*p* value	p*	OR	95% CI (UL)	95% CI (LL)	*t*	SE(*t*)	MAF	*N* calls at locus	Ocular AA	Urogenital AA
168,413	A (61.54)	G (93.33)	*CTA_0156*	CDS	5E-05	1E-04	21.56	6.11	137.25	4.07	0.75	0.50	0.04	H	R
95,863	A (60.47)	G (86.67)	*CTA_0087*	CDS	7E-05	1E-04	9.56	3.47	33.86	3.98	0.57	0.49	0.02	E	G
785,083	A (62.20)	G (96.67)	*pbpB*	CDS	2E-04	1E-04	45.92	9.34	831.41	3.70	1.03	0.49	0.05	I	V
777,345	A (58.59)	G (96.67)	*karG*	CDS	3E-04	1E-04	40.71	8.29	736.79	3.59	1.03	0.47	0.04	Y	H
156,982	C (51.54)	T (90.00)	*oppA_1*	CDS	4E-04	1E-04	9.44	3.13	40.92	3.54	0.63	0.43	0.02	V	I
637,206	A (56.59)	C (96.67)	*sctR*	CDS	5E-04	1E-04	36.25	7.39	655.80	3.48	1.03	0.45	0.03	K	Q
157,069	A (51.54)	G (86.67)	*oppA_1*	CDS	7E-04	3E-04	6.81	2.48	24.09	3.39	0.57	0.44	0.02	S	P
367,095	C (60.77)	T (73.33)	*CTA_0348*	CDS	1E-03	1E-03	4.23	1.81	10.82	3.20	0.45	0.46	0.01	T	I
544,233	A (61.54)	G (73.33)	*CTA_0510*	CDS	1E-03	3E-04	4.23	1.81	10.82	3.20	0.45	0.46	0.02	R	G
954,865	A (59.69)	G (73.33)	*pmpD*	CDS	2E-03	1E-04	4.04	1.73	10.33	3.10	0.45	0.46	0.04	E	G
969,418	C (59.06)	T (73.33)	*sucD*	CDS	2E-03	1E-04	3.94	1.68	10.07	3.04	0.45	0.46	0.03	T	I
544,610	A (61.54)	G (70.00)	*atoS*	CDS	3E-03	1E-03	3.59	1.56	8.85	2.92	0.44	0.45	0.01	D	G
543,548	T (60.63)	C (70.00)	*CTA_0508*	CDS	5E-03	1E-04	0.29	0.12	0.67	−2.83	0.44	0.45	0.06	F	S
969,583	T (58.73)	C (70.00)	*sucD*	CDS	7E-03	1E-04	0.30	0.12	0.70	−2.72	0.44	0.46	0.04	L	P
44,611	C (60.63)	T (66.67)	*CTA_0043*	CDS	1E-02	1E-04	2.96	1.30	7.10	2.53	0.43	0.45	0.04	A	V
533,906	T (74.62)	C (50.00)	*CTA_0498*	CDS	1E-02	9E-03	0.35	0.15	0.80	−2.51	0.42	0.31	0.01	L	P
295,635	G (61.24)	A (63.33)	*CTA_0284*	CDS	2E-02	1E-04	0.38	0.16	0.86	−2.30	0.42	0.44	0.03	R	K
95,527	C (60.77)	T (60.00)	*CTA_0087*	CDS	5E-02	4E-02	2.24	1.00	5.15	1.94	0.41	0.44	0.01	S	L
413,567	A (60.47)	G (60.00)	*CTA_0391*	CDS	6E-02	1E-04	2.21	0.99	5.08	1.91	0.41	0.44	0.04	V	A
1,027,490	G (58.91)	T (60.00)	*CTA_0948*	CDS	7E-02	1E-04	2.13	0.96	4.91	1.83	0.41	0.45	0.01	P	Q
777,183	T (58.59)	C (60.00)	*karG*	CDS	7E-02	1E-04	0.47	0.21	1.06	−1.80	0.41	0.45	0.04	I	V
168,413	A (61.54)	G (93.33)	*CTA_0156*	CDS	5E-05	1E-04	21.56	6.11	137.2	4.07	0.75	0.50	0.04	H	R
(B)															
SNP position	Reference allele	Alternative allele	Name *A/HAR-13*	CDS/NCR	Strand	p*	*p* value	*t*	SE(*t*)	OR	95% CI (UL)	(LL)	MAF	N calls at locus	
1,028,728	C	T	*pmpE*	CDS	–	0.013	0.011	−2.550	0.555	0.078	0.026	0.232	0.310	7.042	
875,804	C	T	*alaS*	CDS	–	0.024	0.022	−2.298	0.530	0.100	0.036	0.284	0.310	4.225	
939,488	G	A	*glgA*	CDS	–	0.026	0.023	−2.273	0.491	0.103	0.039	0.270	0.479	4.225	
285,610	G	A	*CTA_0273*	CDS	–	0.027	0.034	−2.123	0.526	0.120	0.043	0.336	0.310	4.225	
32,779	G	A	*trmD*	CDS	+	0.032	0.031	−2.160	0.525	0.115	0.041	0.323	0.310	2.817	
465,330	C	G	*yjfH*	CDS	–	0.037	0.042	−2.032	0.519	0.131	0.047	0.362	0.310	1.408	
787,841	A	G	NA	inter	NA	0.038	0.038	−2.074	0.524	0.126	0.045	0.351	0.310	4.225	
827,184	A	G	*CTA_0774*	CDS	+	0.041	0.043	−2.020	0.516	0.133	0.048	0.365	0.310	1.408	
22,049	G	T	*ileS*	CDS	+	0.057	0.050	−1.962	0.505	0.141	0.052	0.378	0.324	4.225	
152,011	G	A	NA	inter	NA	0.058	0.050	−1.964	0.505	0.140	0.052	0.377	0.324	4.225	
710,787	A	C	*CTA_0675*	CDS	–	0.060	0.052	−1.941	0.517	0.144	0.052	0.396	0.310	4.225	
19,085	T	C	NA	inter	NA	0.061	0.060	−1.882	0.530	0.152	0.054	0.430	0.296	5.634	
388,175	G	A	*CTA_0368*	CDS	–	0.061	0.059	−1.889	0.524	0.151	0.054	0.422	0.296	1.408	
696,782	A	T	*rpoD*	CDS	–	0.064	0.062	−1.864	0.511	0.155	0.057	0.422	0.310	1.408	
286,636	C	T	*lgt*	CDS	–	0.065	0.061	−1.876	0.511	0.153	0.056	0.417	0.310	0.000	
930,453	C	T	*mutS*	CDS	–	0.067	0.061	−1.876	0.511	0.153	0.056	0.417	0.310	0.000	
465,525	C	T	*CTA_0439*	CDS	–	0.067	0.062	−1.865	0.472	0.155	0.061	0.391	0.493	1.408	
60,858	G	A	*CTA_0057*	CDS	–	0.068	0.070	−1.813	0.512	0.163	0.060	0.445	0.310	1.408	
835,039	G	A	*CTA_0782*	CDS	–	0.070	0.061	−1.876	0.511	0.153	0.056	0.417	0.310	0.000	
19,005	A	G	NA	inter	NA	0.071	0.071	−1.807	0.525	0.164	0.059	0.459	0.296	2.817	
4554	A	G	*gatB*	CDS	+	0.071	0.070	−1.813	0.512	0.163	0.060	0.445	0.310	1.408	
303,590	C	A	*murE*	CDS	–	0.072	0.061	−1.876	0.511	0.153	0.056	0.417	0.310	0.000	
215,130	C	T	*gyrA_1*	CDS	–	0.072	0.062	−1.864	0.511	0.155	0.057	0.422	0.310	1.408	
806,382	C	T	*CTA_0761*	CDS	+	0.073	0.058	−1.896	0.530	0.150	0.053	0.424	0.296	4.225	
778,783	G	A	*rrf*	CDS	–	0.077	0.075	−1.780	0.502	0.169	0.063	0.451	0.324	2.817	
136,812	G	A	*incF*	CDS	+	0.079	0.075	−1.780	0.502	0.169	0.063	0.451	0.324	2.817	
169,573	G	A	*CTA_0156*	CDS	+	0.082	0.077	−1.771	0.523	0.170	0.061	0.474	0.310	9.859	
956,953	C	T	*pmpD*	CDS	+	0.082	0.072	−1.800	0.523	0.165	0.059	0.461	0.296	2.817	
44,990	A	G	*ruvB*	CDS	+	0.087	0.086	−1.718	0.493	0.179	0.068	0.472	0.338	2.817	
62,140	G	T	*sucA*	CDS	+	0.091	0.078	−1.760	0.502	0.172	0.064	0.461	0.324	5.634	
542,521	G	A	*CTA_0507*	CDS	–	0.092	0.090	−1.696	0.494	0.183	0.070	0.483	0.338	2.817	
181,019	C	A	*CTA_0164*	CDS	–	0.095	0.096	−1.666	0.494	0.189	0.072	0.498	0.338	4.225	
151,156	C	G	*CTA_0140*	CDS	–	0.096	0.077	1.770	0.502	5.871	2.195	15.703	0.324	4.225	
1,028,728	C	A	*pmpE*	CDS	–	0.01	0.011	−2.550	0.555	0.08	0.03	0.23	0.31	13.58%	
1,028,728	C	T	*pmpE*	CDS	–	0.0134	0.0108	−2.5504	0.5550	0.0781	0.0263	0.2317	0.3099	7.0423	
875,804	C	T	*alaS*	CDS	–	0.0242	0.0216	−2.2981	0.5295	0.1005	0.0356	0.2836	0.3099	4.2254	
939,488	G	A	*glgA*	CDS	–	0.0259	0.0230	−2.2727	0.4906	0.1030	0.0394	0.2695	0.4789	4.2254	
285,610	G	A	*CTA_0273*	CDS	–	0.0269	0.0338	−2.1226	0.5264	0.1197	0.0427	0.3359	0.3099	4.2254	
32,779	G	A	*trmD*	CDS	+	0.0318	0.0308	−2.1596	0.5248	0.1154	0.0412	0.3227	0.3099	2.8169	
465,330	C	G	*yjfH*	CDS	–	0.0370	0.0422	−2.0315	0.5187	0.1311	0.0474	0.3625	0.3099	1.4085	
787,841	A	G	NA	inter	NA	0.0377	0.0381	−2.0742	0.5236	0.1257	0.0450	0.3506	0.3099	4.2254	
827,184	A	G	*CTA_0774*	CDS	+	0.0413	0.0433	−2.0203	0.5164	0.1326	0.0482	0.3648	0.3099	1.4085	
22,049	G	T	*ileS*	CDS	+	0.0568	0.0497	−1.9624	0.5052	0.1405	0.0522	0.3782	0.3239	4.2254	
152,011	G	A	NA	inter	NA	0.0578	0.0495	−1.9642	0.5051	0.1403	0.0521	0.3775	0.3239	4.2254	
710,787	A	C	*CTA_0675*	CDS	–	0.0605	0.0523	−1.9409	0.5174	0.1436	0.0521	0.3958	0.3099	4.2254	
19,085	T	C	NA	inter	NA	0.0608	0.0598	−1.8819	0.5298	0.1523	0.0539	0.4302	0.2958	5.6338	
388,175	G	A	*CTA_0368*	CDS	–	0.0610	0.0589	−1.8889	0.5238	0.1512	0.0542	0.4222	0.2958	1.4085	
696,782	A	T	*rpoD*	CDS	–	0.0638	0.0623	−1.8643	0.5114	0.1550	0.0569	0.4223	0.3099	1.4085	
286,636	C	T	*lgt*	CDS	–	0.0654	0.0606	−1.8764	0.5113	0.1531	0.0562	0.4172	0.3099	0.0000	
930,453	C	T	*mutS*	CDS	–	0.0668	0.0606	−1.8764	0.5113	0.1531	0.0562	0.4172	0.3099	0.0000	
465,525	C	T	*CTA_0439*	CDS	–	0.0670	0.0622	−1.8650	0.4719	0.1549	0.0614	0.3905	0.4930	1.4085	
60,858	G	A	*CTA_0057*	CDS	–	0.0684	0.0698	−1.8134	0.5121	0.1631	0.0598	0.4450	0.3099	1.4085	
835,039	G	A	*CTA_0782*	CDS	–	0.0700	0.0606	−1.8764	0.5113	0.1531	0.0562	0.4172	0.3099	0.0000	
19,005	A	G	NA	inter	NA	0.0710	0.0707	−1.8074	0.5254	0.1641	0.0586	0.4595	0.2958	2.8169	
4554	A	G	*gatB*	CDS	+	0.0713	0.0698	−1.8134	0.5121	0.1631	0.0598	0.4450	0.3099	1.4085	
303,590	C	A	*murE*	CDS	–	0.0718	0.0606	−1.8764	0.5113	0.1531	0.0562	0.4172	0.3099	0.0000	
215,130	C	T	*gyrA_1*	CDS	–	0.0722	0.0623	−1.8643	0.5114	0.1550	0.0569	0.4223	0.3099	1.4085	
806,382	C	T	*CTA_0761*	CDS	+	0.0726	0.0580	−1.8960	0.5297	0.1502	0.0532	0.4241	0.2958	4.2254	
778,783	G	A	*rrf*	CDS	–	0.0767	0.0751	−1.7797	0.5021	0.1687	0.0630	0.4514	0.3239	2.8169	
136,812	G	A	*incF*	CDS	+	0.0792	0.0751	−1.7797	0.5021	0.1687	0.0630	0.4514	0.3239	2.8169	
169,573	G	A	*CTA_0156*	CDS	+	0.0821	0.0765	−1.7712	0.5227	0.1701	0.0611	0.4740	0.3099	9.8592	
956,953	C	T	*pmpD*	CDS	+	0.0823	0.0719	−1.7998	0.5226	0.1653	0.0594	0.4605	0.2958	2.8169	
44,990	A	G	*ruvB*	CDS	+	0.0871	0.0858	−1.7181	0.4932	0.1794	0.0682	0.4717	0.3380	2.8169	
62,140	G	T	*sucA*	CDS	+	0.0914	0.0784	−1.7601	0.5024	0.1720	0.0643	0.4605	0.3239	5.6338	
542,521	G	A	*CTA_0507*	CDS	–	0.0916	0.0899	−1.6960	0.4940	0.1834	0.0696	0.4830	0.3380	2.8169	
181,019	C	A	*CTA_0164*	CDS	–	0.0953	0.0958	−1.6656	0.4940	0.1891	0.0718	0.4979	0.3380	4.2254	
151,156	C	G	*CTA_0140*	CDS	–	0.0955	0.0767	1.7701	0.5019	5.8714	2.1953	15.7035	0.3239	4.2254	

(a) Ocular localization-associated non-synonymous SNPs (*p* value < 0.1). Position of the SNPs and name of the impacted gene are from the *Ct A/HAR-13* (GenBank accession number NC_007429) genome. ‘Allele percentage’ is the percentage of each group where the given allele was present. ‘CDS/NCR’ identifies whether the SNP was in a coding or non-coding region. ‘p*’ indicates *p* values from 100,024 simulations indicating genome-wide significance at p* < 0.05. ‘*t*’ is the *t* statistic; SE(*t*) is the standard error of the *t* statistic. ‘OR’ is the adjusted odds ratio (derived from the *t* statistic). ‘95% CI’ = 95% confidence interval of the OR.; ‘UL’ upper limit, ‘LL’ lower limit. ‘MAF’ is the minor allele frequency. ‘*N* calls at locus’ is the proportion of isolates which had no base called. ‘AA’ is the amino acid coded for

(b) Disease severity-associated SNPs (*p* value < 0.1). Disease severity is defined by a composite in vivo conjunctival phenotype derived using principal component analysis using ocular *C. trachomatis* load and conjunctival inflammatory (P) score (using the modified FPC (follicles, papillary hypertrophy, conjunctival scarring) trachoma grading system [39]). ‘Reference allele’ indicates the reference allele on *Ct A/HAR-13* (GenBank accession number NC_007429). ‘CDS/NCR’ identifies whether the SNP was in a coding, non-coding or intergenic region. ‘p*’ = permuted *p* value after 100,024 simulations indicating genome-wide significance at p* < 0.05. ‘*t*’ is the *t* statistic; SE(*t*) is the standard error of the *t* statistic. ‘OR’ is the adjusted odds ratio (derived from the *t* statistic). ‘95% CI’ = 95% confidence interval of the OR; ‘UL’ upper limit, ‘LL’ lower limit. ‘MAF’ is the minor allele frequency. ‘*N* calls at locus’ is the proportion of isolates which had no base called

### Markers of disease severity in ocular *C. trachomatis* infection

Using permutation-based re-sampling methods, eight SNPs were found to be significantly associated with disease severity (Fig. 5b). Seven of these are in coding regions (relative to *Ct A/HAR-13*). Five are present at nucleotide positions 465,330 (OR = 0.13, p* = 0.037), 32,779 (OR = 0.12, p* = 0.032), 875,804 (OR = 0.10, p* = 0.024), 939,488 (OR = 0.10, p* = 0.026) and 1,028,728 (OR = 0.08, p* = 0.013) (where p* is the permuted *p* value with a genome-wide threshold of 0.05) representing synonymous codon changes within the genes *yjfH*, *trmD*, *alaS, glgA* and *pmpE* respectively. Three further genome-wide significant synonymous SNPs were present at positions 827,184 (OR = 0.3, p* = 0.041) within the predicted coding sequence (CDS) *CTA0744,* 285,610 (OR = 0.12, p* = 0.027) within *CTA0273* and 787,841 (OR = 0.13, p* = 0.043) in the intergenic region between loci *CTA0744–CTA0745* (Table 2b and Additional file 10: Figure S10).

## Discussion

This collection of clinical ocular *Ct* WGS from a single trachoma-endemic population to be characterized has enabled us to describe the population diversity of naturally occurring *Ct* in a treatment-naïve population. We used detailed clinical grading combined with microbial quantitation to perform a GWAS and investigated associations between *Ct* polymorphisms with ocular localization and disease severity in trachoma.

Unlike the recently published Australian *Ct* sequences [10], all Bijagós sequences clustered as expected within the T2 ocular clade derived from a urogenital ancestor [59, 61], each with loci typically associated with ocular tissue localization (*trpA* and *tarP*). Although the Bijagós sequences conform to the classical ocular genotype, the phylogenetic data show greater than expected diversity compared to historical reference strains of ocular *Ct* [4] and a population of clinical ocular *Ct* sequences obtained from cultured clinical conjunctival swab specimens collected from another African trachoma-endemic population [64] (Additional file 4: Figure S4). Our use of direct WGS from clinical samples reveals the natural diversity of a population-based collection of endemic treatment-naïve ocular *Ct* infections. This diversity may indicate genome-wide selection for advantageous mutations as demonstrated in other pathogens [79] or simply the naturally diverse circulation of endemic treatment-naïve ocular *Ct*.

The apparent village-level clustering provides new evidence that WGS has the necessary molecular resolution to fully investigate *Ct* transmission. Although the number of sequences from each village was very small, overall *Ct* genomic diversity supports our hypothesis of ongoing or recent transmission, since diversity requires mutation, recombination and gene flow. The data from this study demonstrate such mutation and indicate that WGS data may be useful in defining transmission networks and developing transmission maps, which have not been adequately defined using alternative *Ct* genotyping systems. Whole genome mapping has previously been shown to be a useful tool in the analysis of outbreaks and bacterial pathogen transmission [80, 81] and thus has multiple potential applications in epidemiological analysis and transmission studies. However, greater numbers of sequences per village are required to validate this finding.

Such diversity is likely to be representative of recombination present in *Ct* [82]. Genome-wide recombination was common and widespread within these sequences. Extensive recombination has been noted in previous studies and is thought to be a source of diversification with possible interstrain recombination [4, 82]. Recombination may represent fixation of recombination in regions that are under diversifying selection pressure [4].

Recently, a handful of bacterial GWASs have provided insight into the genetic basis of bacterial host preference, antibiotic resistance and virulence [83–88]. Until now, most inferences regarding disease-modifying virulence factors in chlamydial infection have been derived from a limited number of comparative genomic studies where only a few virulence factors were associated with disease severity. Chlamydial genomic association data have previously been used to highlight genes potentially involved in pathoadaptation [10, 89] and tissue localization [90].

In the current GWAS we found 21 genome-wide significant non-synonymous SNPs associated with ocular localization and eight genome-wide significant synonymous SNPs associated with disease severity.

Confidence that new SNPs identified in the ocular localization GWAS are candidate markers of pathoadaptation is supported by the observation that half of the SNPs identified have previously been described as polymorphic or recombinant within *Ct* and the ocular serovars [8, 91–93].

In support of the hypothesis that early events in infection and intracellular growth are crucial events in *Ct* survival and pathogenicity, we identified SNPs within genes that are expressed from the beginning of the chlamydial developmental cycle including *CTA0156* (encoding early endosomal antigen 1 (EEA1) [73]), *CTA0498* (encoding translocated actin-recruiting phosphoprotein (*tarP*) [94]) and *CTA0884* (encoding polymorphic membrane protein D (PmpD) [95]), which have identified roles in entry to and initial interactions with host cells.

Two of the four genes containing multiple non-synonymous SNPs (*karG* and *sucD*) are involved in ATP metabolism and, more generally, chlamydial metabolism. Two of the genes with multiple synonymous mutations (*ruvB* and *CTA_0284*) are also involved in metabolism. Growth rates are known to vary significantly between biovars. The developmental cycle in ocular serovars is substantially longer than that in genital serovars [96]. These genes and the identified SNPs may therefore be important in the differential growth and development of *Ct* serovars. This is supported by the downregulation of *sucD* expression during in vitro persistence. Slower growth in ocular strains occurs primarily in the entry and early stages of differentiation, which may also indicate the role of previously described genes involved in entry into cells.

The eight disease severity-associated SNPs are within less well-characterized genes. Apart from *pmpE*, the remaining genes identified in this study have been shown to be relatively conserved [90]. This suggests that these SNPs may be important in ocular *Ct* pathogenesis, rather than in longer term chlamydial evolution. Three of these genes are putative *Ct* virulence factors, with functions in nutrient acquisition (*glgA* [24, 28, 97]), host-cell adhesion (*pmpE* [98]) and response to IFNγ-induced stress (*trmD* [73]). Homologues of *alaS* [99, 100] and *CTA0273* [101, 102] are known virulence factors in related Gram-negative bacteria, suggesting that these genes are potentially important in *Ct* pathogenesis.

Transcriptome analysis of chlamydial growth in vitro has shown that there is highly upregulated gene expression of *trmD* (encoding a transfer RNA (tRNA) methyltransferase) associated with growth in the presence of IFNγ, thought to be important in the maintenance of chlamydial infection [73]. *yjfH* (renamed *rlmB*) is phylogenetically related to the TrmD family and encodes the protein RlmB, which is important for the synthesis and assembly of the components of the ribosome [103]. In *Escherichia coli*, *Haemophilus influenzae* and *Mycoplasma genitalium*, RlmB catalyses the methylation of guanosine 2251 in 23S ribosomal RNA (rRNA), which is of importance in peptidyl tRNA recognition but is not essential for bacterial growth [103, 104]. *alaS* encodes a tRNA ligase of the class II aminoacyl tRNA synthetase family involved in cytoplasmic protein biosynthesis. It is not known to have virulence associations in chlamydial infection, but has been described as a component of a virulence operon in *Haemophilus ducreyi* [99] and *H. influenzae* [100]. The CDS *CTA0273* encodes a predicted inner membrane protein translocase component of the autotransporter YidC, an inner membrane insertase important in virulence in *E. coli* [101] and *Streptococcus mutans* [102]. Our study suggests that these loci may be important in disease severity and host-pathogen interactions in chlamydial infection. A summary of available literature for these key ocular localization and disease severity-associated SNPs is tabulated in Additional file 11: Figure S11. We cannot speculate further on the effect of these polymorphisms on expression. It is possible that the synonymous disease severity-associated SNPs are markers in linkage for disease-causing alleles that were not included in the final GWAS analysis. For both analyses, further mechanistic studies are required to establish causality and validity and to fully understand the nature of the associations presented.

Though we were intrinsically limited to those cases where infection was detectable and from which we were able to obtain *Ct* WGS data, our population-based treatment-naïve sample attempts to provide a representative picture of what is observed in ocular *Ct* infection. We acknowledge that there may be *Ct* genotypes that are cleared by the immune system such that we do not capture them in a cross-sectional study. We are limited to the small sample size in this study, but attempt to address the issues of statistical power and multiple testing by using a bi-dimensional conjunctival phenotype and permutation-based multivariable regression analysis. To date, many published microbial GWASs have sample sizes under 500 [105], including several key studies examining virulence [84] and drug resistance [85] in *Staphylococcus aureus* with sample sizes of 75 and 90 respectively.

## Conclusions

The potential of bacterial GWASs has only recently been realized, and despite the limitations with sample size, their use to study *Ct* in this way is particularly important, since in vitro models are intrinsically difficult to develop, and it has not been possible to study urogenital *Ct* in the same way due to the lack of a clearly defined in vivo disease phenotype. The genomic markers identified in this study provide important direction for validation through in vitro functional studies and a unique opportunity to understand host-pathogen interactions likely to be important in *Ct* pathogenesis in humans. The greater than expected diversity within this population of naturally circulating ocular *Ct* and the clustering at village level demonstrate the potential utility of WGS in epidemiological and clinical studies. This will enable us to understand transmission in both ocular and urogenital *Ct* infection and will have significant public health implications in preventing and eliminating chlamydial disease in humans.

## Additional files

Additional file 1: **Figure S1.** Histogram and density plot showing log-transformed *C. trachomatis* load (*omcB* copies/swab) data. (PDF 111 kb)

Additional file 2: **Figure S2.** Detailed summary of whole genome sequence (WGS) data quality control of Bijagós *Chlamydia trachomatis* sequences. (PDF 76 kb)

Additional file 3: **Figure S3.** R Script used for (A) tissue localization and (B) disease severity *Chlamydia trachomatis* GWAS. (PDF 180 kb)

Additional file 4: **Figure S4.** Maximum likelihood reconstruction of whole genome phylogeny of *Chlamydia trachomatis* sequences examined in the tissue localization analysis. (PDF 357 kb)

Additional file 5: **Figure S5.** Maximum likelihood reconstruction of the *ompA* (*CTA0742*) phylogeny. (PDF 450 kb)

Additional file 6: **Figure S6.** Recombination present across Bijagós *Chlamydia trachomatis* genome sequences using the pairwise homoplasy index (Phi) and the site-wise log likelihood support for the best-scoring maximum likelihood tree. (PDF 153 kb)

Additional file 7: **Figure S7.** Tyrosine repeat regions and actin-binding domains in *tarP* (*CTA0948*) and polymorphisms in the *trp* operon (*CTA0182*–*CTA0186*) (*trpR, trpB* and *trpA*) within Bijagós (Bissau-Guinean) ocular *Chlamydia trachomatis* sequences. (PDF 49 kb)

Additional file 8: **Figure S8.** Maximum likelihood reconstruction of phylogeny by polymorphic membrane protein (Pmp) genes A–I. (PDF 1738 kb)

Additional file 9: **Figure S9.** Ocular localization-associated SNPs (*p* value < 0.1). (PDF 150 kb)

Additional file 10: **Figure S10.** SNPs across the *Chlamydia trachomatis* genome associated with disease severity using permutation-based genome-wide association analysis. (PDF 158 kb)

Additional file 11: **Figure S11.** Summary of published studies supporting the key ocular localization and disease severity-associated SNPs [106–114]. (PDF 105 kb)

Additional file 12: **Figure S12.** European Nucleotide Archive (ENA) (European Bioinformatics Institute (EBI)) accession numbers relating to *C. trachomatis* sequence data analysed in this study. (PDF 75 kb)

## Abbreviations

ATP: Adenosine triphosphate
*Ct*: *Chlamydia trachomatis*
ddPCR: Droplet digital PCR
DMEM: Dulbecco’s modified Eagle’s medium
DNA: Deoxyribonucleic acid
EB: Elementary body
FPC: Follicles, papillary hypertrophy, conjunctival scarring
GWAS: Genome-wide association study
indels: Insertions and deletions
LGV: Lymphogranuloma venereum
MAF: Minor allele frequency
MOMP: Major outer membrane protein
NHP: Non-human primate
NSS: Neighbour similarity score
PC: Principal component
PCA: Principal component analysis
PCR: Polymerase chain reaction
SNP: Single nucleotide polymorphism
WGS: Whole genome sequencing
