# Supplementary material for: Population-based analysis of ocular Chlamydia trachomatis in trachoma-endemic West African communities identifies genomic markers of disease severity
Source: Genome Med. 2018 Feb 26;10:15. doi: 10.1186/s13073-018-0521-x (PMC5828069; doi:10.1186/s13073-018-0521-x)
Supplement: Supplementary file 1 — Figure S1. Histogram and density plot showing log-transformed C. trachomatis load (omcB copies/swab) data. (PDF 111 kb) [file 13073_2018_521_MOESM1_ESM.pdf]

Figure S1. Histogram and density plot showing log-transformed *C. trachomatis* load

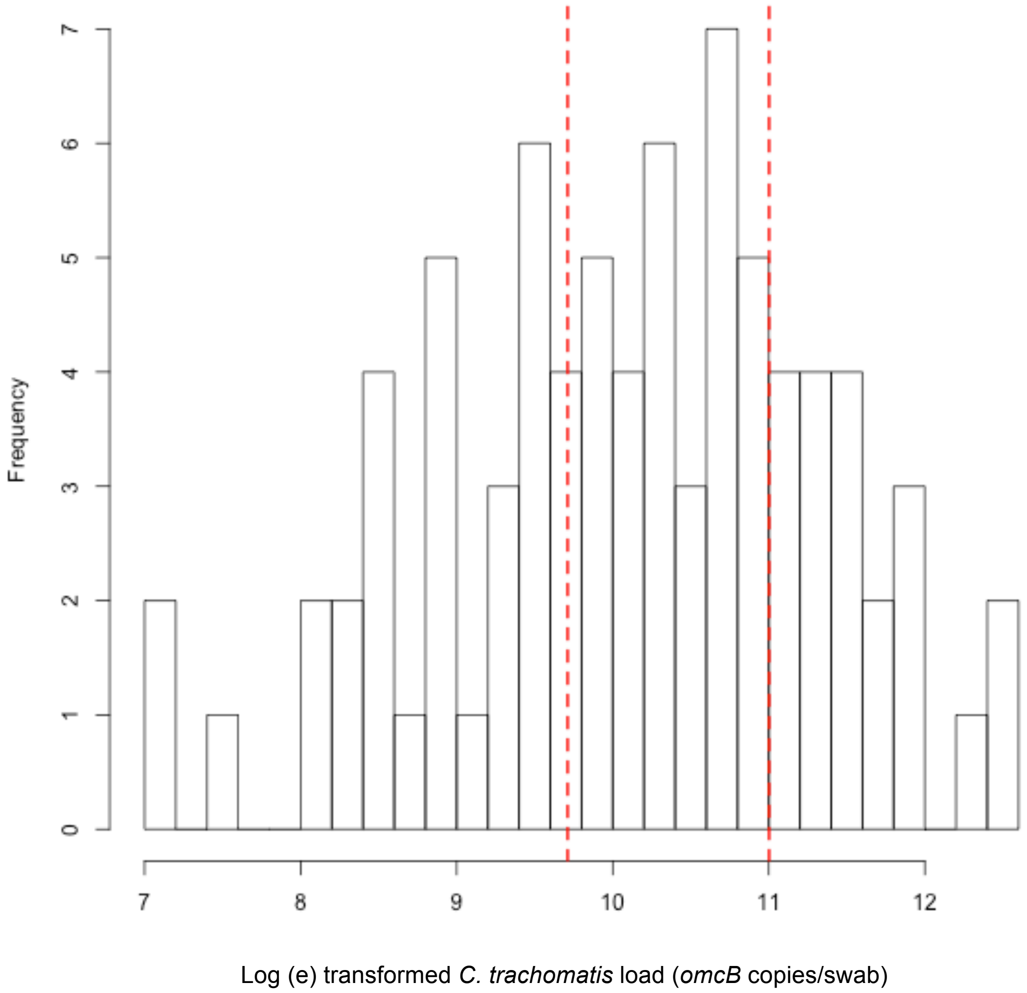

(A) Histogram showing log-transformed *C. trachomatis* load (*omcB* copies/swab) data. Red lines indicate tertiles.

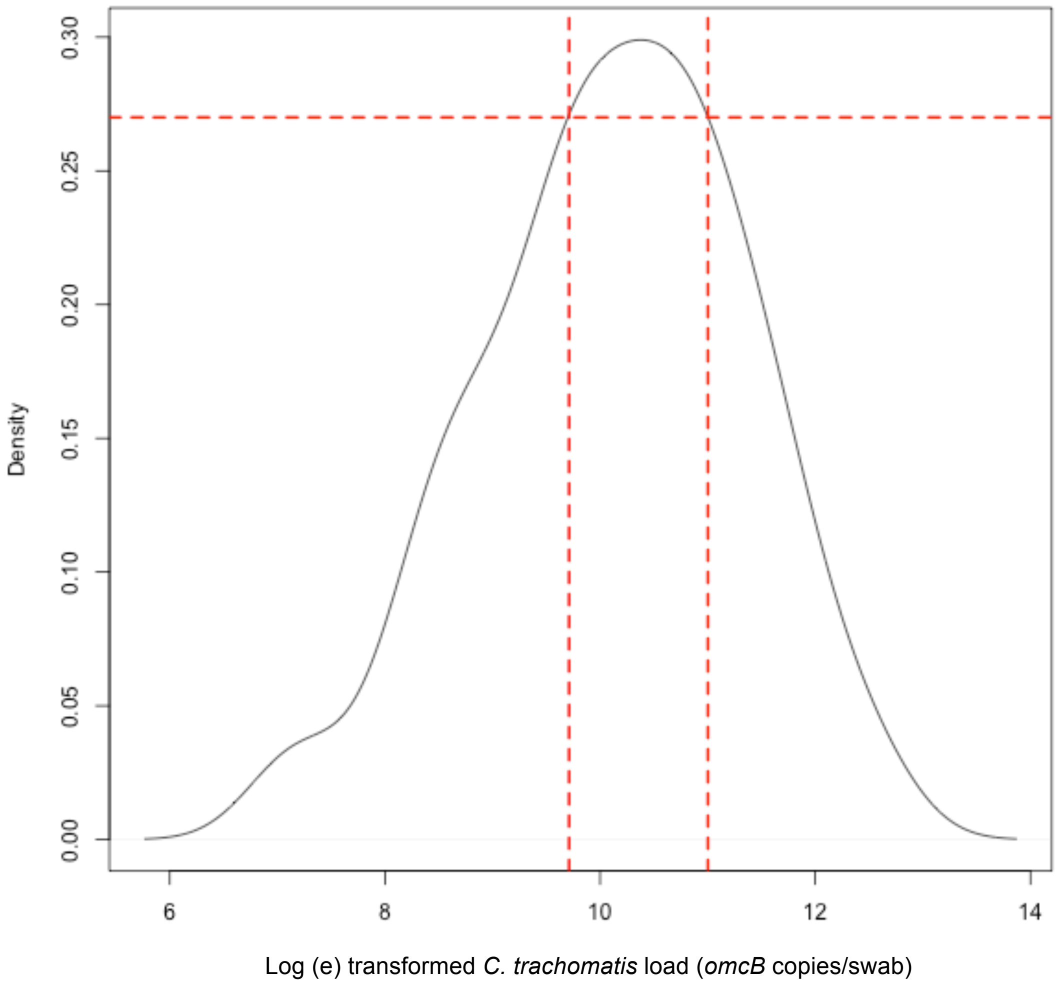

(B) Density plot related to log-transformed *C. trachomatis* load (*omcB* copies/swab). Vertical red lines indicate tertiles as in (A).
