# Supplementary material for: Population-based analysis of ocular Chlamydia trachomatis in trachoma-endemic West African communities identifies genomic markers of disease severity
Source: Genome Med. 2018 Feb 26;10:15. doi: 10.1186/s13073-018-0521-x (PMC5828069; doi:10.1186/s13073-018-0521-x)
Supplement: Supplementary file 2 — Figure S2. Detailed summary of whole genome sequence (WGS) data quality control of Bijagós Chlamydia trachomatis sequences. (PDF 76 kb) [file 13073_2018_521_MOESM2_ESM.pdf]

Figure S2. Detailed summary of whole genome sequence (WGS) data quality control of Bijagós *Chlamydia trachomatis* sequences.

Sequence ID	Total Sequence	Read Length	Average Depth of Coverage	GC%
11152_3_1	5739170	75	764	41
11152_3_10	937544	75	121	41
11152_3_11	150592	75	19	41
11152_3_12	527232	75	68	41
11152_3_13	176129	75	21	41
11152_3_14	12351377	75	1664	41
11152_3_15	1437909	75	191	41
11152_3_16	15354132	75	2065	41
11152_3_17	469098	75	61	41
11152_3_18	346718	75	44	41
11152_3_19	6202868	75	825	41
11152_3_2	57682	75	6	41
11152_3_20	22785576	75	3070	41
11152_3_21	98006	75	11	41
11152_3_22	408768	75	51	41
11152_3_23	22785619	75	3643	41
11152_3_24	10602	75	0	43
11152_3_25	1856962	75	246	41
11152_3_26	20484603	75	2746	41
11152_3_27	12322865	75	1664	41
11152_3_28	1097089	75	143	41
11152_3_29	420126	75	53	41
11152_3_3	403490	75	51	41
11152_3_30	846872	75	107	41
11152_3_31	1343971	75	177	41
11152_3_32	197953	75	24	41
11152_3_33	2886258	75	381	41
11152_3_34	379478	75	48	41
11152_3_35	4275649	75	571	41
11152_3_36	3727749	75	496	41
11152_3_37	161920	75	20	41
11152_3_38	643059	75	81	41
11152_3_39	1836077	75	243	41
11152_3_4	1160431	75	150	41
11152_3_40	1118149	75	145	41
11152_3_41	303059	75	37	41
11152_3_42	778315	75	101	41
11152_3_43	539559	75	70	41
11152_3_44	246224	75	31	41
11152_3_45	109389	75	12	41
11152_3_46	10271	75	0	42

11152_3_47	112277	75	13	41
11152_3_48	474314	75	61	41
11152_3_49	24669	75	1	42
11152_3_5	1770107	75	235	41
11152_3_50	363367	75	46	41
11152_3_51	85619	75	9	41
11152_3_52	169197	75	20	41
11152_3_53	110662	75	12	41
11152_3_54	120025	75	14	41
11152_3_55	457936	75	59	41
11152_3_56	76830	75	8	41
11152_3_57	168168	75	21	41
11152_3_58	261821	75	33	41
11152_3_59	45678	75	4	41
11152_3_6	406036	75	52	41
11152_3_60	105885	75	12	41
11152_3_61	68424	75	7	41
11152_3_62	179007	75	22	41
11152_3_63	142302	75	17	41
11152_3_64	34052	75	3	41
11152_3_65	60498	75	6	41
11152_3_66	360272	75	45	41
11152_3_67	35893	75	3	41
11152_3_68	27323	75	2	41
11152_3_69	99097	75	11	41
11152_3_7	4457616	75	581	41
11152_3_70	207780	75	25	41
11152_3_71	26634	75	2	41
11152_3_72	7015	75	0	43
11152_3_73	63508	75	7	41
11152_3_74	198545	75	24	41
11152_3_75	54261	75	5	41
11152_3_76	3184903	75	417	41
11152_3_77	802297	75	105	41
11152_3_78	136164	75	16	41
11152_3_79	6825	75	0	44
11152_3_8	302624	75	38	41
11152_3_80	7466	75	0	44
11152_3_81	13925	75	0	42
11152_3_82	36312	75	3	42
11152_3_83	42372	75	4	42
11152_3_84	35068	75	3	42
11152_3_85	18141	75	0	42
11152_3_86	18663	75	0	42
11152_3_87	5897	75	0	43
11152_3_88	1235578	75	163	41

11152_3_89	11370	75	0	43
11152_3_9	168278	75	20	41
11152_3_90	8184	75	0	43
11152_3_91	12722	75	0	43
11152_3_92	560194	75	73	41
11152_3_93	16431	75	0	42
11152_3_94	118096	75	14	41
11152_3_95	55338	75	6	41
13108_1_1	71000	75	0	40
13108_1_10	63791	75	1	39
13108_1_11	94163	75	5	39
13108_1_12	142505	75	11	40
13108_1_13	81058	75	2	38
13108_1_14	453228	75	51	41
13108_1_15	318087	75	33	40
13108_1_16	75903	75	1	39
13108_1_17	97270	75	4	39
13108_1_18	78733	75	1	38
13108_1_19	97442	75	4	40
13108_1_2	157753	75	12	40
13108_1_3	94584	75	4	39
13108_1_4	62901	75	0	40
13108_1_5	68048	75	0	39
13108_1_6	58110	75	0	40
13108_1_7	269547	75	27	40
13108_1_8	79463	75	2	39
13108_1_9	208413	75	18	40
8422_8_49	341311	75	39	42
8422_8_50	698440	75	82	42
9471_4_86	1950520	100	287	40
9471_4_87	1921776	100	215	41
9471_4_88	1754289	100	192	40
9471_4_89	2258774	100	119	41
9471_4_90	2143262	100	111	41
9471_4_91	2000674	100	94	41
9471_4_92	2440127	100	85	41
9471_4_93	2199385	100	242	41
9519_1_41	107764	75	13	41
9519_1_44	1014423	75	26	38

Sequences 9471_4_86 through to 9471_4_93 inclusive were obtained using DNA after extraction from inoculae following chlamydial cell culture. All other sequences were obtained directly from clinical samples using SureSelect DNA-bait pre-sequencing enrichment. Sequences were obtained using Illumina paired-end technology (Illumina GAII or Hiseq 2000). All sequences passed on basic sequencing criteria using FastQC software. (www.bioinformatics.babraham.ac.uk/projects/fastqc/).
