# Supplementary material for: Population-based analysis of ocular Chlamydia trachomatis in trachoma-endemic West African communities identifies genomic markers of disease severity
Source: Genome Med. 2018 Feb 26;10:15. doi: 10.1186/s13073-018-0521-x (PMC5828069; doi:10.1186/s13073-018-0521-x)
Supplement: Supplementary file 3 — Figure S3. R Script used for (A) tissue localization and (B) disease severity Chlamydia trachomatis GWAS. (PDF 180 kb) [file 13073_2018_521_MOESM3_ESM.pdf]

Figure S3.
R Script used for (A) tissue localization and (B) disease severity *Chlamydia trachomatis* GWAS

(A)

```
#script for tissue localization genome-wide association study

#read in necessary data

x=read.table("./file_with_genotypes",header=T,sep="\t")

localization=read.table("./file_with_id_and_collection_site[localization]_for_genomes",
header=T,sep="\t")

final_data=as.data.frame(t(x))

final_data[final_data=="N"]=NA

annotate=read.table(paste(path,"file_with_gene_information",sep=""),sep="
",na.strings = ".",header=T,row.names=NULL)

#run generalised linear model, outcome is collection site, variables are genotypes

gmodel=lapply(gvarlist,function(x){


glm(substitute(localization$localization~i,list(i=as.name(x))),data=final_data,family="b
inomial")

})

#get p-values, OR etc

pval=NULL

for (i in 1:1007)

{

  pval[i]=(summary(gmodel[[i]])$coefficients[,4])[2]

}

glm_or=NULL

glm_or2.5=NULL

glm_or97.5=NULL

for (i in 1:1007)
```

```
{
  glm_or[i]=exp(cbind(OR = coef(gmodel[[i]]), confint(gmodel[[i]])))[2]
}
glm_or
for (i in 1:1007)
{
  glm_or2.5[i]=exp(cbind(OR = coef(gmodel[[i]]), confint(gmodel[[i]])))[5]
}
for (i in 1:1007)
{
  glm_or97.5[i]=exp(cbind(OR = coef(gmodel[[i]]), confint(gmodel[[i]])))[8]
}
minor_allele_frequencies<-NA #get MAF values for the remaining SNPs
for (i in 1:dim(final_data)[2])
{
  MAF<-final_data[,i]
  minor_allele_frequencies[i]<-(sort(summary(factor(MAF[which(MAF!="N")])))[1])/
    length(MAF)
}
results=cbind(annotate,pval,glm_or,glm_or2.5,glm_or97.5,minor_allele_frequencies)


#Permutation Test of Association
permuted_pvalue=NULL
for (j in 1:1007)
{
  permutes=data.frame(matrix(ncol = 100000, nrow = 157)) #change 100000 to
required no. of permutations
```

```
  for (i in 1:100000)

  {

    permutes[[i]]=sample(final_data[,j],replace=FALSE)

  }

  varlist=colnames(permutes)

  glmx<- lapply(varlist, function(x) {

    glm(substitute((localization$localization) ~ k, list(k = as.name(x))), data =
permutes,family="binomial")

  })

  multi_pv_0=NULL

  for (i in 1:100000)

  {multi_pv_0[i]=data.frame(summary(glmx[[i]])[12])[2,4]}

  permuted_pvalue[j]=(sum(multi_pv_0<pval[j])+1)/100001

}

final_results=cbind(results,permuted_pvalue)
```

(B)

```
#script for pathogen genome-wide association study
path <- "./"

#PULL In the packages needed for this script

require(qqman)

require(MASS)

require(dplyr)

require(parallel)

#Data Preparation

allelesAT <-
read.table(paste(path,"file_of_genotypes_for_all_isolates",sep=""),header=T) #read
in the genotype data
```

dim(allelesAT) #Get the new dimensions of the genotype data

keepit <- allelesAT$pos #this gets a list of lines in the annotation file for which there is matching SNP data in the genotype.

annotate <- read.table(paste(path,"file_with_gene_information",sep=""),sep=" ",na.strings = ".",header=T,row.names=NULL)#gets the annotation file for the A/HAR13 genome

dim(annotate) #show dimensions of the annotations file

Matching column names with column content

colnames(annotate) <- c("pos","ref","alt_nt1","alt_nt2","alt_nt3","codon_num","aa_ref","aa_alt1","aa_alt2","aa_alt3","gene_id","gene_name","ncr","start","end","strand","gene_coor")#creates a name for each column in the annotation file

Add columns with gene annotations

metadata_newpca <- read.table(paste(path,"file_with_clinical_data_for_isolates",sep=""),header=T,sep="\t") #pull in the clinical data for use as phenotypes etc

#Genotype tables

mixed <- function(x) { sum(1*(nchar(x)>1)) } #function to identify number of SNPs in genotype list where the base call has more than one character, identifies 'heterozygous calls' which are tagged as "0.5" and so have three characters

polys <- function(x) { length(table(x[x != "-" & !is.na(x) & x != "N"])) } #funtion to identify bases that are polymorphic, which are tagged as 2 or more, whilst monomorphic bases are called as 1

qc_calls<-allelesAT[,-c(1:3)] #defines a matrix like object that contains only the genotype data

#change all - calls to N

qc_calls_metadata<-allelesAT[,c(1:3)] #defines a matrix like object that contains only the header data for the SNPs in qc_calls object

rm(allelesAT) #removes the original data file

polycalls <- apply(qc_calls,1,polys) #runs the polys function on qc_calls

print("evidence of polymorphism at this SNP position (number of genotypes)")

print(polycalls) #prints which bases are polymorphic in the data set qc_calls

```
#remove non polymorphic positions from each of the annotate, qc_calls and metadata file

qc_calls<-qc_calls[-which(polycalls==1),]

annotate<-annotate[-which(polycalls==1),]

qc_calls_metadata<-qc_calls_metadata[-which(polycalls==1),]

dim(qc_calls_metadata)

dim(qc_calls)

#look for evidence of heterozygous calls at each SNP

mixecalls <- apply(qc_calls,1,mixed)

print("number of specimens with heterozygosity at this SNP position")

print(mixecalls)

qc_calls<-qc_calls[-which(mixecalls==1),] #remove heterozygous calls from the three main objects

annotate<-annotate[-which(mixecalls==1),]

qc_calls_metadata<-qc_calls_metadata[-which(mixecalls==1),]

#get a transposed version of qc_calls, which will be used in the rest of the analysis for association tests

qc_t<-as.data.frame(t(qc_calls))

for ( i in 1:dim(qc_t)[2])

{

  a<-(which(qc_t[,i]=="-"))

  if(length(a)>0){qc_t[a,i]<-"N"}

}

#get the minor allele frequencies

minor_allele_frequencies<-NA

for (i in 1:dim(qc_t)[2])

{

  MAF<-qc_t[,i]
```

```
  minor_allele_frequencies[i]<-
(sort(summary(factor(MAF[which(MAF!="N")])))[1])/length(MAF)

}

#remove SNPs with MAF <0.25

dim(qc_t)

dim(qc_calls_metadata)

qc_t<-qc_t[,-which(minor_allele_frequencies<0.25)]

qc_calls_metadata<-qc_calls_metadata[-which(minor_allele_frequencies<0.25),]

annotate<-annotate[-which(minor_allele_frequencies<0.25),]

#filter based on the proportion of missing/N calls per SNP

number_of_n_calls_per_snp<-NA

for (i in 1:dim(qc_t)[2]){number_of_n_calls_per_snp[i]<-length(which(qc_t[,i]=="N"))}

summary(number_of_n_calls_per_snp)

annotate$percentage_n_calls_at_locus<-number_of_n_calls_per_snp/dim(qc_t)[1]

#filter based on proportion of missing/N calls per specimen genome-wide

number_calls_are_n_for_specimen<-NA

for (i in 1:dim(qc_t)[1]){number_calls_are_n_for_specimen[i]<-
length(which(qc_t[i,]=="N"))}

summary(number_calls_are_n_for_specimen)

names(number_calls_are_n_for_specimen)<-rownames(qc_t)

#get positions of specimens with more than 25% missing/N calls genome-wide

omit_bad_DNA<-which(number_calls_are_n_for_specimen/dim(qc_t)[2]>0.25)

metadata_newpca<-metadata_newpca[-omit_bad_DNA,]#remove bad specimens
from the metadata

qc_t<-qc_t[-omit_bad_DNA,]#remove bad specimens from the genotype data

#DATA IS NOW READY FOR ASSOCIATION TEST
```

```
#########################

# TESTS OF ASSOCIATION

#########################


#define the output variable for the simple test of association

pval<-NA

t<-NA

t.se<-NA

or=NA

cilow=NA

cihigh=NA

#works through each SNP, runs polr based association test and returns vector of p values in object pval, correcting for age

for(i in 1:dim(qc_t)[2])

{

  genotypes_for_assoc_test<-as.character(qc_t[,i])

  genotypes_for_assoc_test<-gsub(pattern = "N",replacement = NA,x = genotypes_for_assoc_test)

  #the next two lines make the allele corresponding to the reference genome the reference allele for the regression

  xxx=as.character(annotate$ref[i])

  result<-summary(polr(factor(metadata_newpca$PCA1)~relevel(factor(genotypes_for_assoc_test),xxx)+metadata_newpca$age,Hess = T))

  pval[i]<-pnorm(abs(coef(result)[1,3]), lower.tail = FALSE) * 2

  t[i]<-(coef(result)[1,3])

  t.se[i]<-(coef(result)[1,2])

  or[i]<-exp(coef(result)[1,3])

  cilow[i]<-exp(result$coefficients[1,3]-(1.96*result$coefficients[1,2]))
```

```
  cihigh[i]<-exp(result$coefficients[1,3]+(1.96*result$coefficients[1,2]))

}


#get MAF values for the remaining SNPs and paste in to main table of results

minor_allele_frequencies<-NA

for (i in 1:dim(qc_t)[2])

{

  MAF<-qc_t[,i]

  minor_allele_frequencies[i]<-
(sort(summary(factor(MAF[which(MAF!="N")]))))[1])/length(MAF)

}


#put together the results of the basic test

assoc.test.results<-cbind(pval,t,t.se,minor_allele_frequencies)

annotate<-cbind(annotate,assoc.test.results)


#################################

#Permutation Test of Association

################################

true_phenotypes<-metadata_newpca$PCA1 #capture the real phenotypes in a
simple vector for speed

true_pvalue<-pval #capture the real p values in a simple vector for speed

set.seed(100) #set the random seed for making samples.

###################

#define the permutation test

###################
```

```
perm_test<-function(true_phenotypes,true_pvalue,ages) #defining the function in this parenthesis

{

  sim_phenotype<-sample(true_phenotypes,length(true_phenotypes)) #get a randomisation of the phenotypes

  temp_perm_pval<-NA  #make a place to put the put the data

  for(j in 1:dim(qc_t)[2])
  {
    genotypes_for_impute<-as.character(qc_t[,j])
    genotypes_for_impute<-gsub(pattern = "N",replacement = NA,x = genotypes_for_impute)
    xxx=as.character(annotate$ref[i])
    result<-summary(polr(factor(sim_phenotype)~relevel(factor(genotypes_for_impute),xxx)+ages,Hess = T))
    temp_perm_pval[j]<-pnorm(abs(coef(result)[1,3]), lower.tail = FALSE) * 2
  } #do the permutation test of the genotypes

  return(temp_perm_pval)

}
####################
#Run on cluster
set.seed(100)
```

```
numCores <- detectCores()

cl2 <- makePSOCKcluster(rep(x = "localhost",times=numCores))

clusterExport(cl2, c("metadata_newpca","pval","perm_test","qc_t","polr"))



system.time(a<-clusterCall(cl=cl2,fun = function(X) perm_test(true_phenotypes =
metadata_newpca$PCA1,true_pvalue = pval,ages = metadata_newpca$age))) #test
run should return eight randomised results in object a




#this runs lots of permutations, set 1:x where x = nperms/numcores. i.e. for 8 cores
and 1000 perms, use 1:125 for 1000 permutations

system.time(

for(i in 1:12500)

{

  print(i)

  b<-clusterCall(cl=cl2,fun = function(X) perm_test(true_phenotypes =
metadata_newpca$PCA1,true_pvalue = pval,ages = metadata_newpca$age))

  a<-c(a,b)

}

)

stopCluster(cl2) #make sure that this line gets run



#convert the list in to a table

perm_results<-matrix(unlist(a),ncol=dim(qc_t)[2],byrow = F)



###finally compute the permuted P values by finding what proportion of Permuted p
statistics are smaller than the true value of p.

perm_p<-NA

for (i in 1:length(true_pvalue))
```

```
{
  perm_p[i]<-length(which(perm_results[,i]<=true_pvalue[i]))/dim(perm_results)[1]
}


#bind the results table to the perm p values

perm_results_output<-cbind(annotate,perm_p)

#calculate the odds ratio and confidence intervals

perm_results_output$OR<-exp(perm_results_output$t)

perm_results_output$OR_low<-exp(perm_results_output$t-
(1.96*perm_results_output$t.se))

perm_results_output$OR_high<-
exp(perm_results_output$t+(1.96*perm_results_output$t.se))
```

Creative Commons Attribution 4.0 International Public License

By exercising the Licensed Rights (defined below), You accept and agree to be bound by the terms and conditions of this Creative Commons Attribution 4.0 International Public License ("Public License"). To the extent this Public License may be interpreted as a contract, You are granted the Licensed Rights in consideration of Your acceptance of these terms and conditions, and the Licensor grants You such rights in consideration of benefits the Licensor receives from making the Licensed Material available under these terms and conditions.

Section 1 – Definitions.

a. **Adapted Material** means material subject to Copyright and Similar Rights that is derived from or based upon the Licensed Material and in which the Licensed Material is translated, altered, arranged, transformed, or otherwise modified in a manner requiring permission under the Copyright and Similar Rights held by the Licensor. For purposes of this Public License, where the Licensed Material is a musical work, performance, or sound recording, Adapted Material is always produced where the Licensed Material is synched in timed relation with a moving image.

b. **Adapter's License** means the license You apply to Your Copyright and Similar Rights in Your contributions to Adapted Material in accordance with the terms and conditions of this Public License.

c. **Copyright and Similar Rights** means copyright and/or similar rights closely related to copyright including, without limitation, performance, broadcast, sound recording, and Sui Generis Database Rights, without regard to how the rights are labeled or categorized. For purposes of this Public License, the rights specified in Section 2(b)(1)-(2) are not Copyright and Similar Rights.

d. **Effective Technological Measures** means those measures that, in the absence of proper authority, may not be circumvented under laws fulfilling obligations under Article 11 of the WIPO Copyright Treaty adopted on December 20, 1996, and/or similar international agreements.

e. **Exceptions and Limitations** means fair use, fair dealing, and/or any other exception or limitation to Copyright and Similar Rights that applies to Your use of the Licensed Material.

f. **Licensed Material** means the artistic or literary work, database, or other material to which the Licensor applied this Public License.

g. **Licensed Rights** means the rights granted to You subject to the terms and conditions of this Public License, which are limited to all Copyright and Similar Rights that apply to Your use of the Licensed Material and that the Licensor has authority to license.

h. **Licensor** means the individual(s) or entity(ies) granting rights under this Public License.

i. **Share** means to provide material to the public by any means or process that requires permission under the Licensed Rights, such as reproduction, public display, public performance, distribution, dissemination, communication, or importation, and to make material available to the public including in ways that members of the public may access the material from a place and at a time individually chosen by them.

j. **Sui Generis Database Rights** means rights other than copyright resulting from Directive 96/9/EC of the European Parliament and of the Council of 11 March 1996 on the legal protection of databases, as amended and/or succeeded, as well as other essentially equivalent rights anywhere in the world.

k. **You** means the individual or entity exercising the Licensed Rights under this Public License. **Your** has a corresponding meaning.

Section 2 – Scope.

a. **License grant**.

   1. Subject to the terms and conditions of this Public License, the Licensor hereby grants You a worldwide, royalty-free, non-sublicensable, non-exclusive, irrevocable license to exercise the Licensed Rights in the Licensed Material to:

      A. reproduce and Share the Licensed Material, in whole or in part; and

      B. produce, reproduce, and Share Adapted Material.

   2. Exceptions and Limitations. For the avoidance of doubt, where Exceptions and Limitations apply to Your use, this Public License does not apply, and You do not need to comply with its terms and conditions.

   3. Term. The term of this Public License is specified in Section 6(a).

   4. Media and formats; technical modifications allowed. The Licensor authorizes You to exercise the Licensed Rights in all media and formats whether now known or hereafter created, and to make technical modifications necessary to do so. The Licensor waives and/or agrees not to assert any right or authority to forbid You from making technical modifications necessary to exercise the Licensed Rights, including technical modifications necessary to circumvent Effective Technological Measures. For purposes of this Public License,

simply making modifications authorized by this Section 2(a)(4) never produces Adapted Material.

5. Downstream recipients.

    A. Offer from the Licensor – Licensed Material. Every recipient of the Licensed Material automatically receives an offer from the Licensor to exercise the Licensed Rights under the terms and conditions of this Public License.

    B. No downstream restrictions. You may not offer or impose any additional or different terms or conditions on, or apply any Effective Technological Measures to, the Licensed Material if doing so restricts exercise of the Licensed Rights by any recipient of the Licensed Material.

6. No endorsement. Nothing in this Public License constitutes or may be construed as permission to assert or imply that You are, or that Your use of the Licensed Material is, connected with, or sponsored, endorsed, or granted official status by, the Licensor or others designated to receive attribution as provided in Section 3(a)(1)(A)(i).

b. **Other rights**.

1. Moral rights, such as the right of integrity, are not licensed under this Public License, nor are publicity, privacy, and/or other similar personality rights; however, to the extent possible, the Licensor waives and/or agrees not to assert any such rights held by the Licensor to the limited extent necessary to allow You to exercise the Licensed Rights, but not otherwise.

2. Patent and trademark rights are not licensed under this Public License.

3. To the extent possible, the Licensor waives any right to collect royalties from You for the exercise of the Licensed Rights, whether directly or through a collecting society under any voluntary or waivable statutory or compulsory licensing scheme. In all other cases the Licensor expressly reserves any right to collect such royalties.

Section 3 – License Conditions.

Your exercise of the Licensed Rights is expressly made subject to the following conditions.

a. **Attribution**.

1. If You Share the Licensed Material (including in modified form), You must:

    A. retain the following if it is supplied by the Licensor with the Licensed Material:

        i. identification of the creator(s) of the Licensed Material and any others designated to receive attribution, in any reasonable manner requested by the Licensor (including by pseudonym if designated);

        ii. a copyright notice;

        iii. a notice that refers to this Public License;

        iv. a notice that refers to the disclaimer of warranties;

        v. a URI or hyperlink to the Licensed Material to the extent reasonably practicable;

B. indicate if You modified the Licensed Material and retain an indication of any previous modifications; and

C. indicate the Licensed Material is licensed under this Public License, and include the text of, or the URI or hyperlink to, this Public License.

2. You may satisfy the conditions in Section 3(a)(1) in any reasonable manner based on the medium, means, and context in which You Share the Licensed Material. For example, it may be reasonable to satisfy the conditions by providing a URI or hyperlink to a resource that includes the required information.
3. If requested by the Licensor, You must remove any of the information required by Section 3(a)(1)(A) to the extent reasonably practicable.
4. If You Share Adapted Material You produce, the Adapter's License You apply must not prevent recipients of the Adapted Material from complying with this Public License.

Section 4 – Sui Generis Database Rights.

Where the Licensed Rights include Sui Generis Database Rights that apply to Your use of the Licensed Material:

a. for the avoidance of doubt, Section 2(a)(1) grants You the right to extract, reuse, reproduce, and Share all or a substantial portion of the contents of the database;

b. if You include all or a substantial portion of the database contents in a database in which You have Sui Generis Database Rights, then the database in which You have Sui Generis Database Rights (but not its individual contents) is Adapted Material; and

c. You must comply with the conditions in Section 3(a) if You Share all or a substantial portion of the contents of the database.

For the avoidance of doubt, this Section 4 supplements and does not replace Your obligations under this Public License where the Licensed Rights include other Copyright and Similar Rights.

Section 5 – Disclaimer of Warranties and Limitation of Liability.

a. **Unless otherwise separately undertaken by the Licensor, to the extent possible, the Licensor offers the Licensed Material as-is and as-available, and makes no representations or warranties of any kind concerning the Licensed Material, whether express, implied, statutory, or other. This includes, without limitation, warranties of title, merchantability, fitness for a particular purpose, non-infringement, absence of latent or other defects, accuracy, or the presence or absence of errors, whether or not known or discoverable. Where disclaimers of warranties are not allowed in full or in part, this disclaimer may not apply to You.**

b. **To the extent possible, in no event will the Licensor be liable to You on any legal theory (including, without limitation, negligence) or otherwise for any direct, special, indirect, incidental, consequential, punitive, exemplary, or other losses, costs, expenses, or damages arising out of this Public License or use of the Licensed Material, even if the Licensor has been advised of the possibility of such losses, costs, expenses, or damages. Where a limitation of liability is not allowed in full or in part, this limitation may not apply to You.**

c. The disclaimer of warranties and limitation of liability provided above shall be interpreted in a manner that, to the extent possible, most closely approximates an absolute disclaimer and waiver of all liability.

Section 6 – Term and Termination.

a. This Public License applies for the term of the Copyright and Similar Rights licensed here. However, if You fail to comply with this Public License, then Your rights under this Public License terminate automatically.
b. Where Your right to use the Licensed Material has terminated under Section 6(a), it reinstates:
   1. automatically as of the date the violation is cured, provided it is cured within 30 days of Your discovery of the violation; or
   2. upon express reinstatement by the Licensor.

   For the avoidance of doubt, this Section 6(b) does not affect any right the Licensor may have to seek remedies for Your violations of this Public License.
c. For the avoidance of doubt, the Licensor may also offer the Licensed Material under separate terms or conditions or stop distributing the Licensed Material at any time; however, doing so will not terminate this Public License.
d. Sections 1, 5, 6, 7, and 8 survive termination of this Public License.

Section 7 – Other Terms and Conditions.

a. The Licensor shall not be bound by any additional or different terms or conditions communicated by You unless expressly agreed.
b. Any arrangements, understandings, or agreements regarding the Licensed Material not stated herein are separate from and independent of the terms and conditions of this Public License.

Section 8 – Interpretation.

a. For the avoidance of doubt, this Public License does not, and shall not be interpreted to, reduce, limit, restrict, or impose conditions on any use of the Licensed Material that could lawfully be made without permission under this Public License.
b. To the extent possible, if any provision of this Public License is deemed unenforceable, it shall be automatically reformed to the minimum extent necessary to make it enforceable. If the provision cannot be reformed, it shall be severed from this Public License without affecting the enforceability of the remaining terms and conditions.
c. No term or condition of this Public License will be waived and no failure to comply consented to unless expressly agreed to by the Licensor.
d. Nothing in this Public License constitutes or may be interpreted as a limitation upon, or waiver of, any privileges and immunities that apply to the Licensor or You, including from the legal processes of any jurisdiction or authority.
