# Supplementary material for: Population-based analysis of ocular Chlamydia trachomatis in trachoma-endemic West African communities identifies genomic markers of disease severity
Source: Genome Med. 2018 Feb 26;10:15. doi: 10.1186/s13073-018-0521-x (PMC5828069; doi:10.1186/s13073-018-0521-x)
Supplement: Supplementary file 4 — Figure S4. Maximum likelihood reconstruction of whole genome phylogeny of Chlamydia trachomatis sequences examined in the tissue localization analysis. (PDF 357 kb) [file 13073_2018_521_MOESM4_ESM.pdf]

Figure S4. Maximum likelihood reconstruction of whole-genome phylogeny of *Chlamydia trachomatis* sequences examined in the tissue localization analysis.

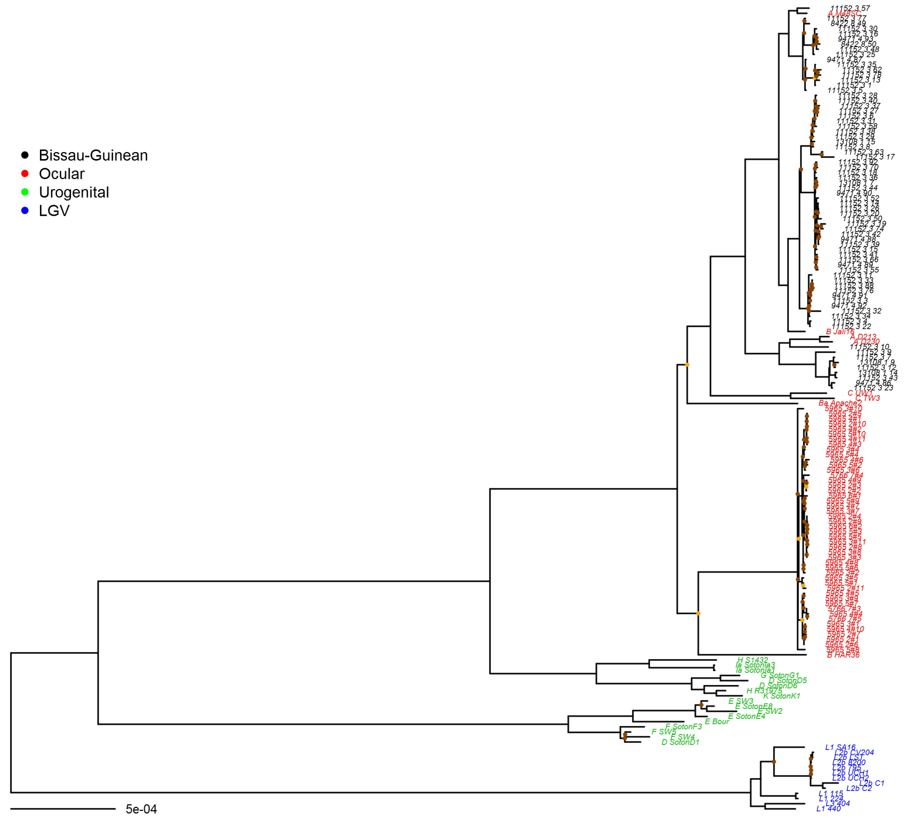

Maximum likelihood reconstruction of the whole-genome phylogeny of 81 *Ct* sequences from the Bijagós Islands, 48 *Ct* sequences from Rombo and 38 *Ct* reference strains. Bijagós *Ct* sequences (n=81) were mapped to *Ct A/HAR-13* using SAMtools [55]. SNPs were called as described by Harris et al. [4]. Phylogenies were computed with RAxML [62] from a variable sites alignment using a GTR+gamma model and are midpoint rooted. The scale bar indicates evolutionary distance. Bijagós *Ct* sequences in this study are coloured BLACK and reference strains are coloured by tissue localization (RED=Ocular, GREEN=Urogenital, BLUE=LGV). Branches are supported by > 90% of 1000 bootstrap replicates. Branches supported by 80-90% (ORANGE) and < 80% (BROWN) bootstrap replicates are indicated.
