# Supplementary material for: Population-based analysis of ocular Chlamydia trachomatis in trachoma-endemic West African communities identifies genomic markers of disease severity
Source: Genome Med. 2018 Feb 26;10:15. doi: 10.1186/s13073-018-0521-x (PMC5828069; doi:10.1186/s13073-018-0521-x)
Supplement: Supplementary file 6 — Figure S6. Recombination present across Bijagós Chlamydia trachomatis genome sequences using the pairwise homoplasy index (Phi) and the site-wise log likelihood support for the best-scoring maximum likelihood tree. (PDF 153 kb) [file 13073_2018_521_MOESM6_ESM.pdf]

Figure S6. Recombination present across Bijagós *Chlamydia trachomatis* genome sequences

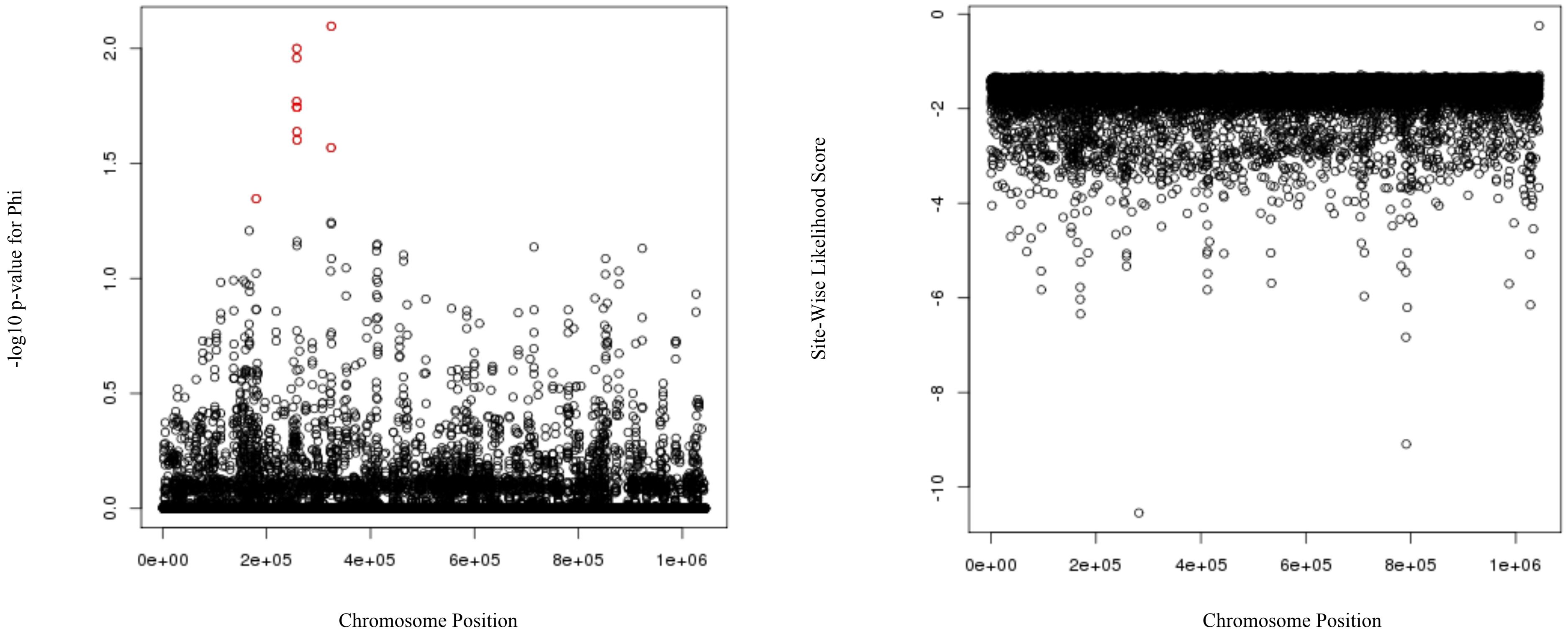

(A) (B)

Widespread recombination with statistically significant hotspots (indicated in red) using (A) the pairwise homoplasy index (Phi) and (B) the site-wise log likelihood support for the best-scoring maximum likelihood tree are demonstrated along the *Chlamydia trachomatis* genome (using all SNPs (n=1034) in sequences with greater than 10x coverage (n=81)). The site-wise log likelihood support for the tree structure calculates the alignment at each nucleotide position and calculates a likelihood score. In the trees constructed for our data, there is no clear region where there is lack of support for the tree based on these measure.
