# Supplementary material for: Population-based analysis of ocular Chlamydia trachomatis in trachoma-endemic West African communities identifies genomic markers of disease severity
Source: Genome Med. 2018 Feb 26;10:15. doi: 10.1186/s13073-018-0521-x (PMC5828069; doi:10.1186/s13073-018-0521-x)
Supplement: Supplementary file 7 — Figure S7. Tyrosine repeat regions and actin-binding domains in tarP (CTA0948) and polymorphisms in the trp operon (CTA0182–CTA0186) (trpR, trpB and trpA) within Bijagós (Bissau-Guinean) ocular Chlamydia trachomatis sequences. (PDF 49 kb) [file 13073_2018_521_MOESM7_ESM.pdf]

Figure S7. Tyrosine Repeat Regions and Actin-Binding Domains in *TARP (CTA0948)* and polymorphisms in the *trp operon (CTA0182-CTA0186) (trpR, trpB and trpA)* within Bijagós (Bissau-Guinean) ocular *Chlamydia trachomatis* sequences.

The trpA mutation is consistent in all ocular *C. trachomatis* sequences. Variability in *trpB/R* mutations is anticipated.

**TARP (*CTA0948*)**		
	**Actin-Binding Domains (ABD)**	
	Three	Four
**Tyrosine Repeat Regions (TRR)**		
3(4,4,4)	20	61
**Trp Operon (*CTA0182-CTA0186*)**		
	**Full-length**	**Truncated**
trpR (*CTA0182*)	67	14
trpB (*CTA0184/0185*)	74	7
trpA (*CTA0186*)	0	81

Ocular *Ct* strains possess more ABD and fewer TRR. Ocular *Ct* strains are expected have between 1-3 TRR and 4 (occasionally 2) ABD. The *Ct* sequences included in this analysis contain the expected ocular lineage-specific 3-4 TRR, and where coverage is adequate, 4 ABD are present.
