# Supplementary material for: Population-based analysis of ocular Chlamydia trachomatis in trachoma-endemic West African communities identifies genomic markers of disease severity
Source: Genome Med. 2018 Feb 26;10:15. doi: 10.1186/s13073-018-0521-x (PMC5828069; doi:10.1186/s13073-018-0521-x)

Figure S8. Maximum likelihood reconstruction of phylogeny by polymorphic membrane protein genes A-I. Trees are midpoint rooted. The scale bar indicates evolutionary distance. Bijagós *Ct* sequences are coloured BLACK, ocular *Ct* reference sequences in RED, urogenital *Ct* reference sequences in GREEN and LGV reference sequences in BLUE. Branches are supported by > 90% of 1000 bootstrap replicates. Branches supported by 80-90% *(ORANGE)* and < 80% *(BROWN)* bootstrap replicates are indicated.

Maximum likelihood reconstruction of the phylogeny *pmpA* (*CTA0447*)

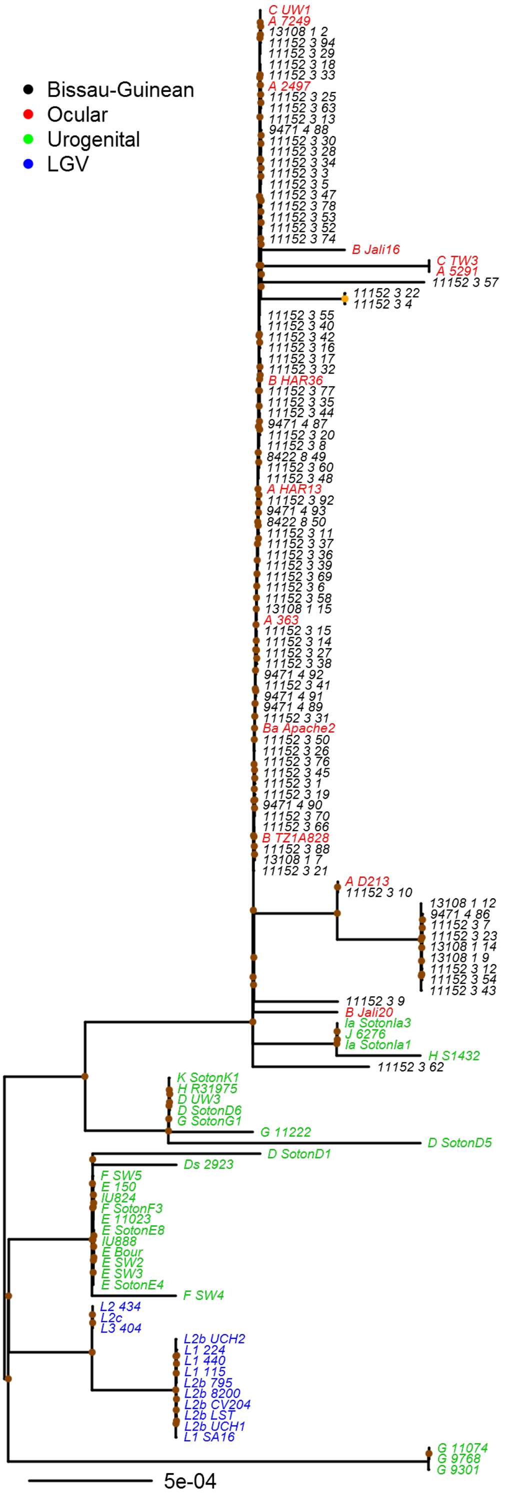

Maximum likelihood reconstruction of the phylogeny *pmpB* (*CTA0448*)

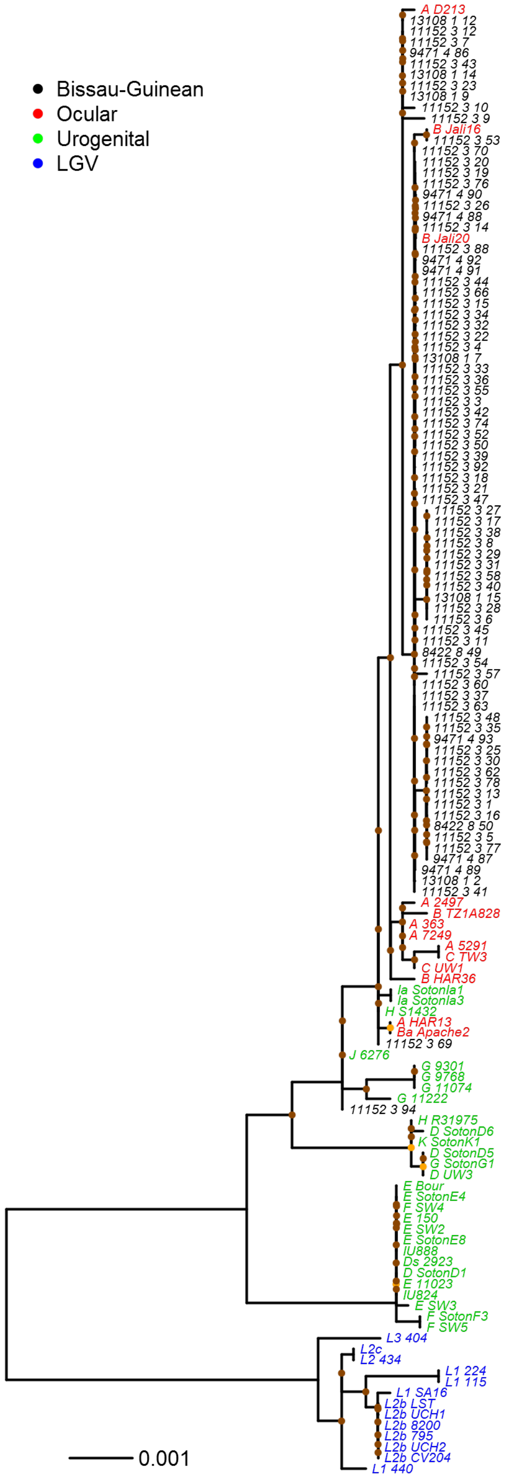

Maximum likelihood reconstruction of the phylogeny *pmpC* (*CTA0449*)

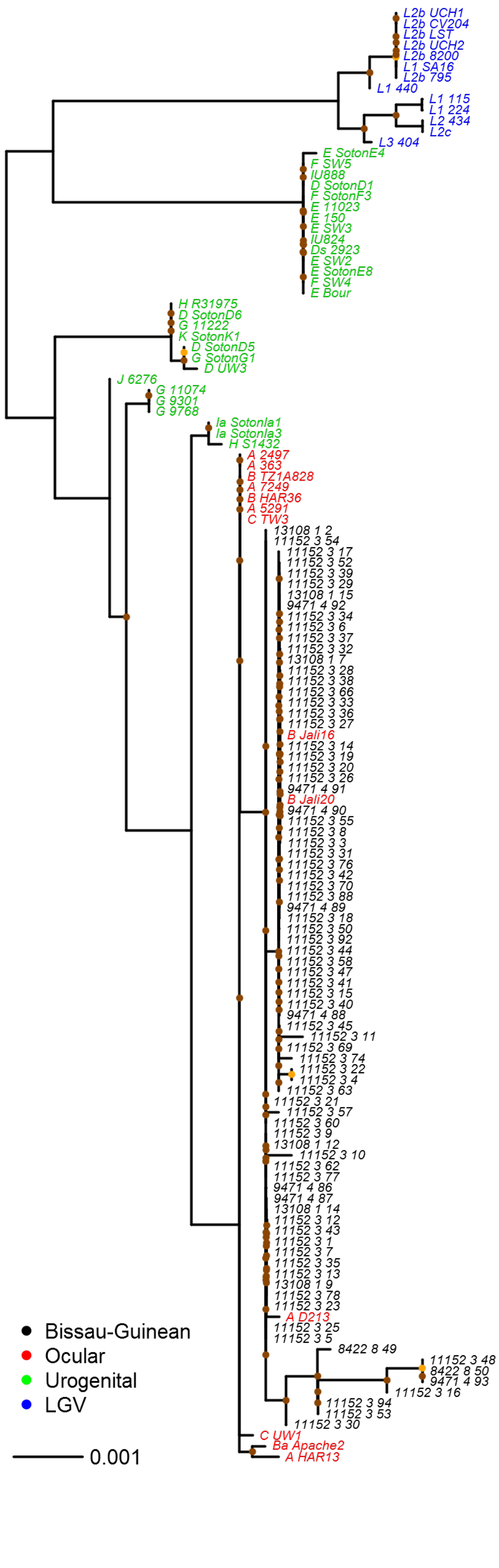

Maximum likelihood reconstruction of the phylogeny *pmpD* (*CTA0884*)

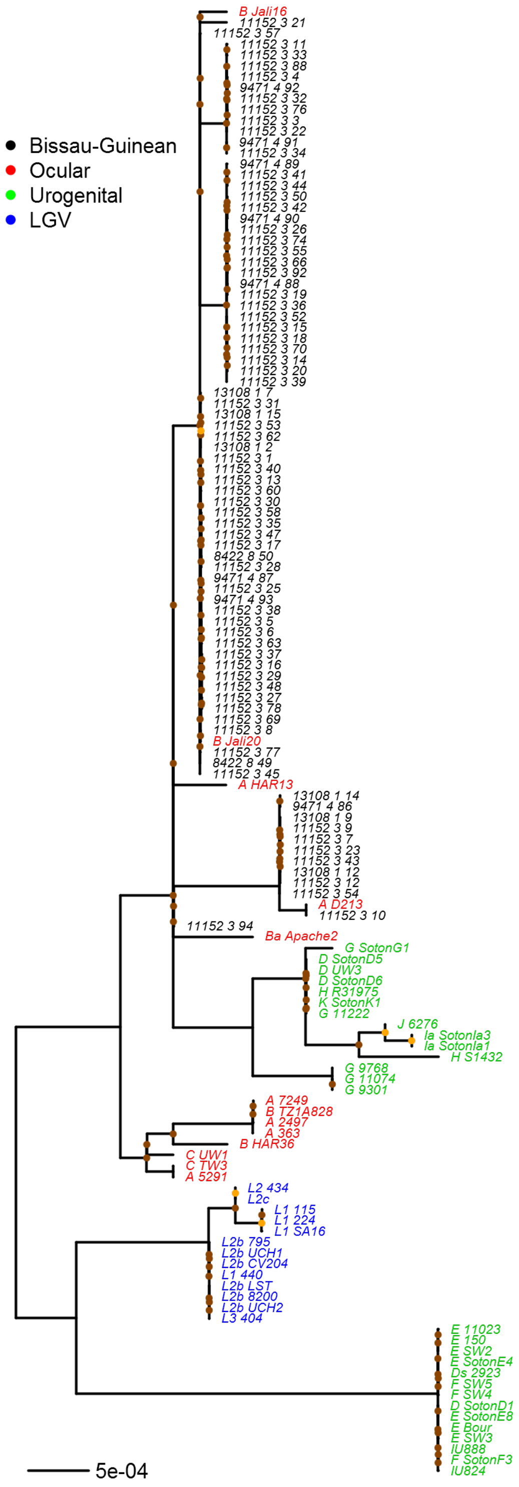

Maximum likelihood reconstruction of the phylogeny *pmpE* (*CTA0949*)

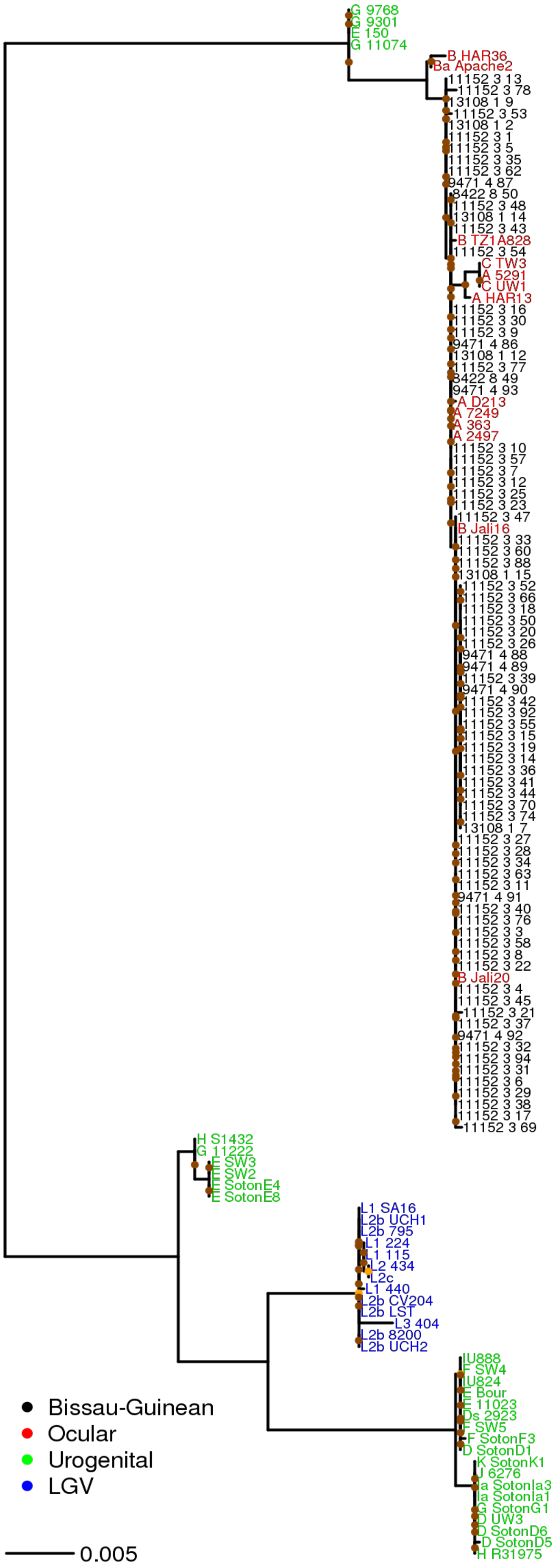

Maximum likelihood reconstruction of the phylogeny *pmpF* (*CTA0950*)

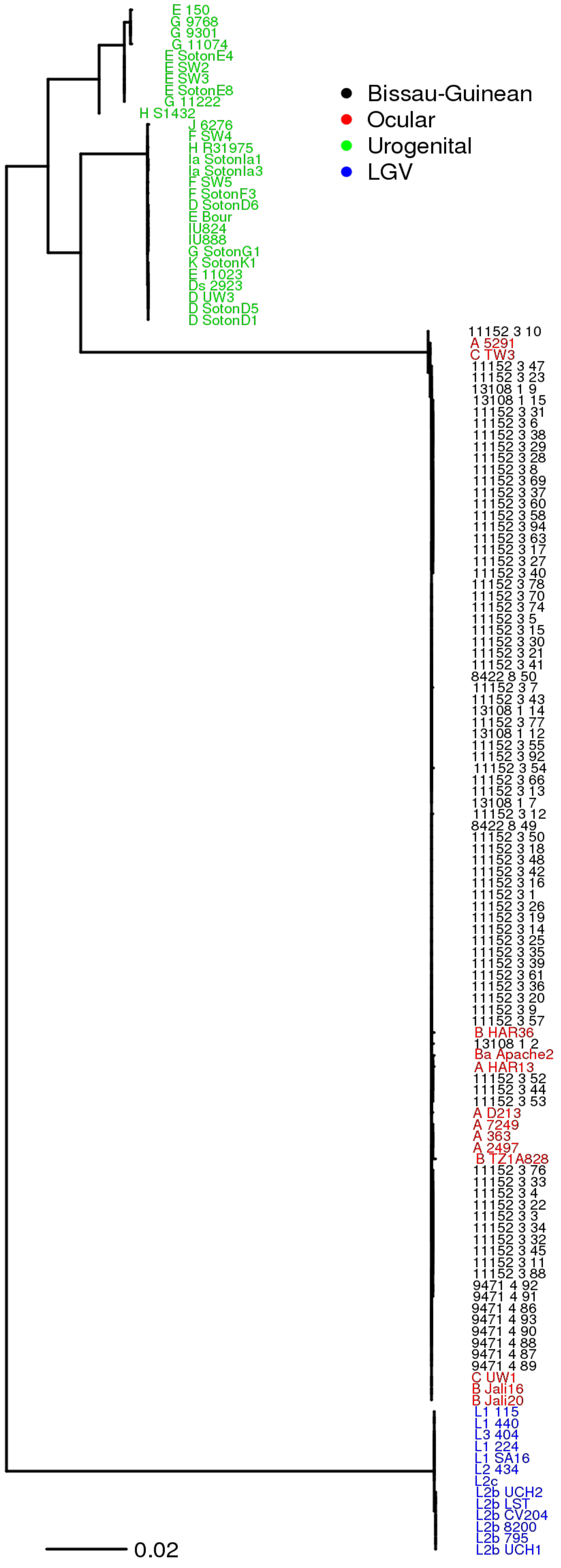

Maximum likelihood reconstruction of the phylogeny pmpG *(*CTA0951*)*

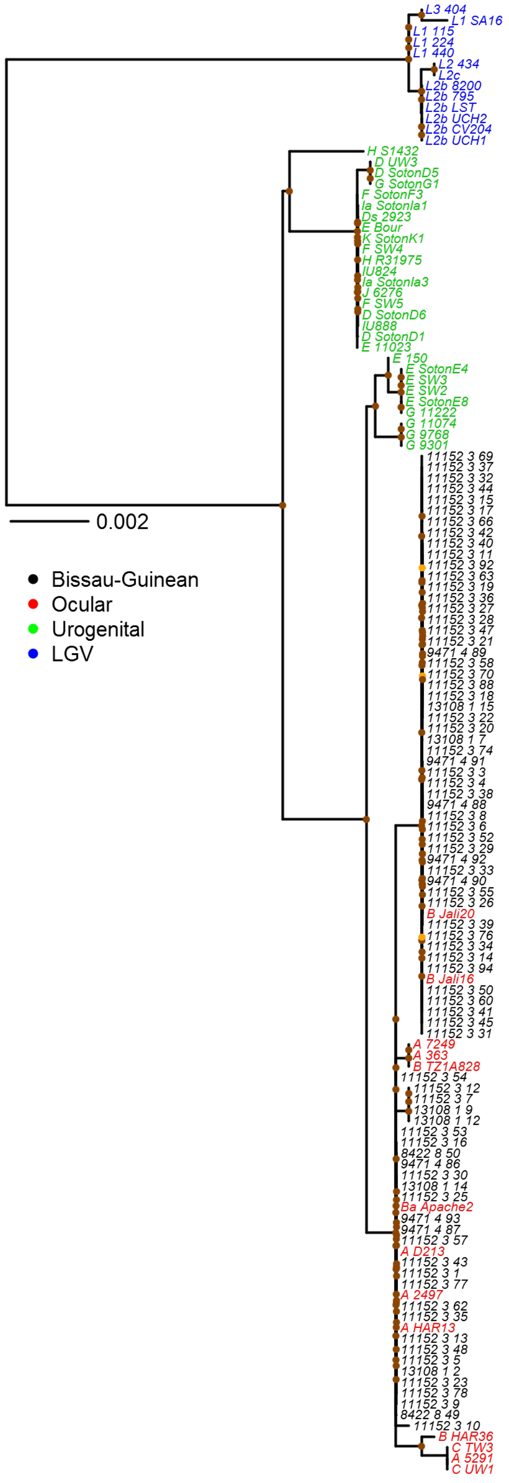

Maximum likelihood reconstruction of the phylogeny *pmpH* (*CTA0952*)

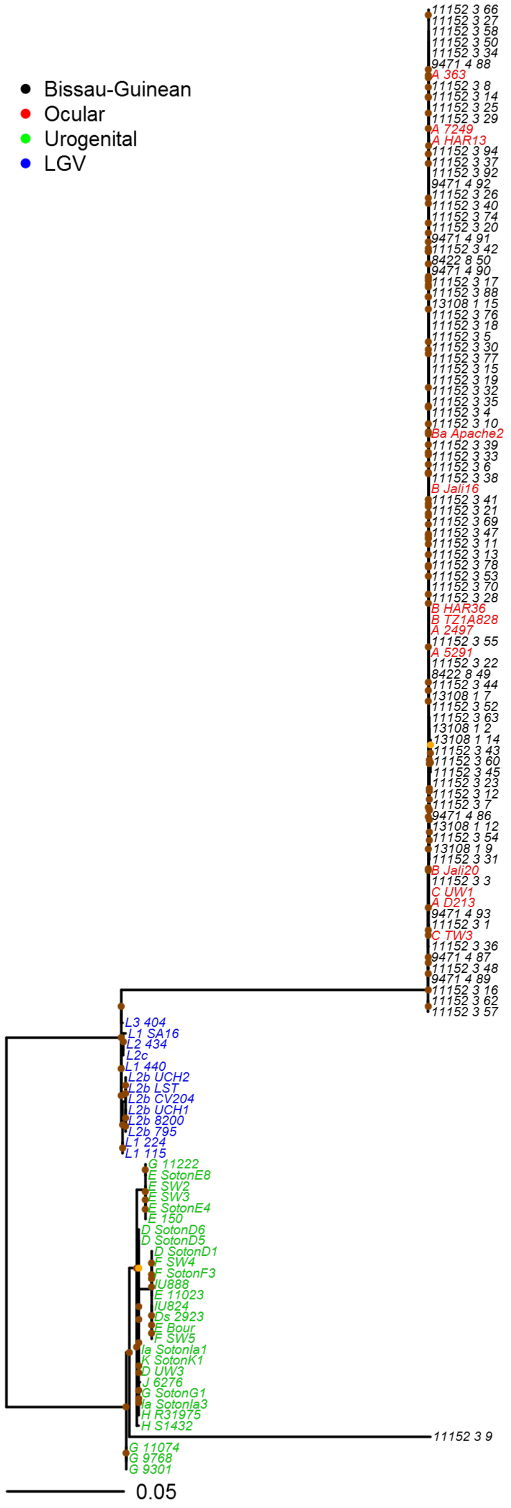

Maximum likelihood reconstruction of the phylogeny *pmpI* (*CTA0954*)

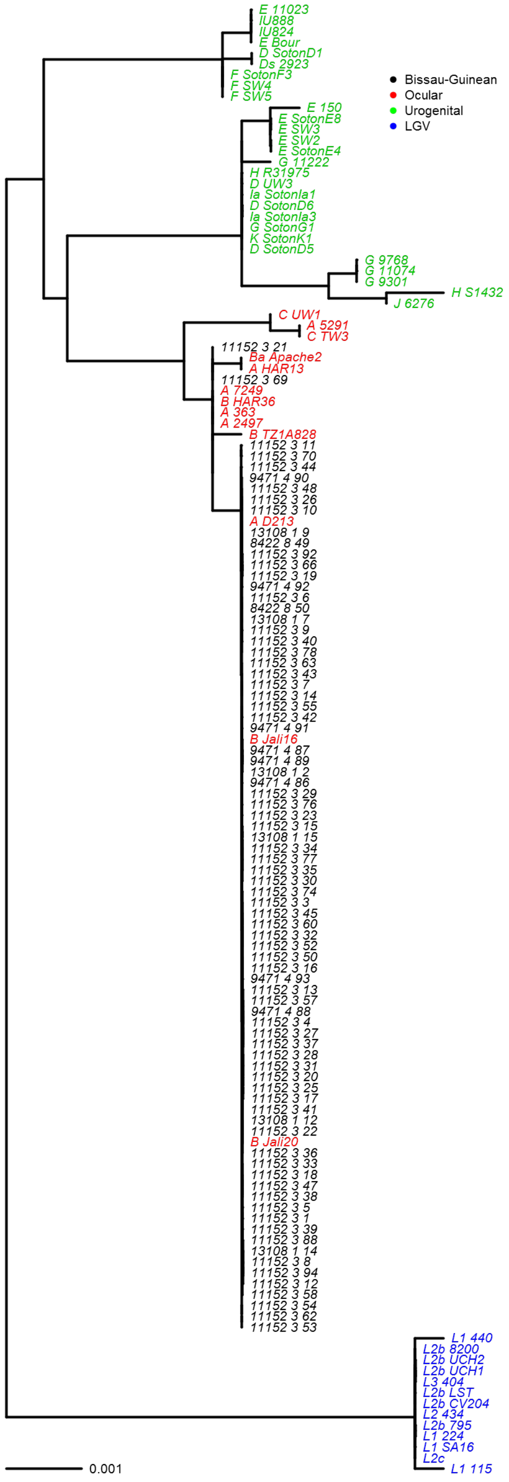

Supplement: Supplementary file 8 — Figure S8. Maximum likelihood reconstruction of phylogeny by polymorphic membrane protein (Pmp) genes A–I. (PDF 1738 kb) [file 13073_2018_521_MOESM8_ESM.pdf]
