# Supplementary material for: Population-based analysis of ocular Chlamydia trachomatis in trachoma-endemic West African communities identifies genomic markers of disease severity
Source: Genome Med. 2018 Feb 26;10:15. doi: 10.1186/s13073-018-0521-x (PMC5828069; doi:10.1186/s13073-018-0521-x)
Supplement: Supplementary file 9 — Figure S9. Ocular localization-associated SNPs (p value < 0.1). (PDF 150 kb) [file 13073_2018_521_MOESM9_ESM.pdf]

Figure S9. Ocular localization-associated SNPs (*p-value < 0.1*). Position of the SNPs and name of the impacted are from the *Ct A/HAR13* genome (GenBank Accession Number NC_007429). ‘Allele Percentage’ is the percentage within each group with the given allele. ‘CDS/NCR’ identifies whether the SNP was in a coding or non-coding region. ‘P*’ indicates p-values from 100,024 simulations indicating genome wide significance at *p*<0.05*. ‘MAF’ is the minor allele frequency. ‘N Calls at Locus’ is the proportion of isolates which had no base called. ‘AA’ is the amino acid coded for.

POSITION	OCULAR ALLELE (%)	UROGENITAL ALLELE (%)	NAME A/HAR13	CDS/NCR	P-VALUE	P*	OR	95% CI		T	SE(T)	MAF	N CALLS AT LOCUS	OCULAR AA	UROGENITAL AA
**781312**	G (81.60)	A (75.00)	NA	inter	8E-08	1E-04	0.07	0.02	0.18	-5.36	0.50	0.27	0.09	NA	NA
**45001**	T (60.94)	C (90.00)	ruvB	CDS	4E-05	1E-04	0.07	0.02	0.22	-4.10	0.64	0.49	0.05	I	I
**168413**	A (61.54)	G (93.33)	CTA_0156	CDS	5E-05	1E-04	21.56	6.11	137.25	4.07	0.75	0.50	0.04	H	R
**95863**	A (60.47)	G (86.67)	CTA_0087	CDS	7E-05	1E-04	9.56	3.47	33.86	3.98	0.57	0.49	0.02	E	G
**779741**	T (54.87)	A (90.00)	tsf	CDS	2E-04	1E-04	0.09	0.02	0.28	-3.73	0.64	0.40	0.16	A	A
**432897**	G (58.46)	A (83.33)	NA	inter	2E-04	1E-04	0.14	0.05	0.37	-3.72	0.52	0.50	0.04	NA	NA
**785083**	A (62.20)	G (96.67)	pbpB	CDS	2E-04	1E-04	45.92	9.34	831.41	3.70	1.03	0.49	0.05	I	V
**164996**	C (61.54)	T (80.00)	dnlJ	CDS	2E-04	2E-04	6.16	2.49	17.59	3.70	0.49	0.47	0.02	H	H
**169146**	A (61.54)	G (96.67)	CTA_0156	CDS	2E-04	2E-04	44.66	9.10	808.28	3.68	1.03	0.50	0.02	K	K
**778452**	A (59.52)	G (96.55)	rrf	CDS	3E-04	1E-04	40.88	8.30	740.52	3.59	1.03	0.47	0.06	R	R
**777345**	A (58.59)	G (96.67)	karG	CDS	3E-04	1E-04	40.71	8.29	736.79	3.59	1.03	0.47	0.04	Y	H
**156982**	C (51.54)	T (90.00)	oppA_1	CDS	4E-04	1E-04	9.44	3.13	40.92	3.54	0.63	0.43	0.02	V	I
**778240**	T (56.41)	G (83.33)	NA	inter	4E-04	1E-04	0.16	0.05	0.41	-3.54	0.52	0.44	0.13	NA	NA
**637206**	A (56.59)	C (96.67)	sctR	CDS	5E-04	1E-04	36.25	7.39	655.80	3.48	1.03	0.45	0.03	K	Q
**779114**	T (59.52)	A (96.00)	pyrH	CDS	6E-04	1E-04	0.03	0.00	0.14	-3.43	1.04	0.47	0.10	P	P
**157069**	A (51.54)	G (86.67)	oppA_1	CDS	7E-04	3E-04	6.81	2.48	24.09	3.39	0.57	0.44	0.02	S	P
**964591**	A (59.69)	G (76.67)	NA	inter	8E-04	1E-04	4.83	2.02	12.95	3.36	0.47	0.47	0.04	NA	NA
**797602**	T (59.20)	C (76.67)	NA	inter	1E-03	1E-04	0.22	0.08	0.52	-3.24	0.47	0.47	0.09	NA	NA
**362134**	T (61.90)	C (73.33)	rplA	CDS	1E-03	1E-04	0.23	0.09	0.55	-3.22	0.45	0.45	0.04	K	K
**295438**	A (61.54)	G (73.33)	CTA_0284	CDS	1E-03	7E-04	4.23	1.81	10.82	3.20	0.45	0.46	0.02	L	L

**367095**	C (60.77)	T (73.33)	CTA_0348	CDS	1E-03	1E-03	4.23	1.81	10.82	3.20	0.45	0.46	0.01	T	I
**544233**	A (61.54)	G (73.33)	CTA_0510	CDS	1E-03	3E-04	4.23	1.81	10.82	3.20	0.45	0.46	0.02	R	G
**324804**	A (61.24)	G (73.33)	CTA_0310	CDS	2E-03	1E-04	4.18	1.79	10.69	3.17	0.45	0.46	0.03	L	L
**44815**	G (60.63)	A (73.33)	ruvB	CDS	2E-03	1E-04	0.25	0.10	0.58	-3.11	0.45	0.46	0.06	Q	Q
**954865**	A (59.69)	G (73.33)	pmpD	CDS	2E-03	1E-04	4.04	1.73	10.33	3.10	0.45	0.46	0.04	E	G
**969434**	C (59.69)	T (73.33)	sucD	CDS	2E-03	1E-04	4.04	1.73	10.33	3.10	0.45	0.46	0.02	S	S
**768118**	G (62.79)	A (70.00)	sctN	CDS	2E-03	1E-04	0.26	0.11	0.61	-3.04	0.44	0.44	0.03	L	L
**969418**	C (59.06)	T (73.33)	sucD	CDS	2E-03	1E-04	3.94	1.68	10.07	3.04	0.45	0.46	0.03	T	I
**881787**	A (63.08)	G (66.67)	NA	inter	3E-03	4E-03	3.52	1.55	8.46	2.93	0.43	0.42	0.02	NA	NA
**965628**	G (60.94)	A (70.00)	typP_2.2	CDS	3E-03	1E-04	0.28	0.11	0.64	-2.93	0.44	0.45	0.03	G	G
**295529**	T (61.54)	C (70.00)	CTA_0284	CDS	3E-03	3E-03	0.28	0.11	0.64	-2.92	0.44	0.45	0.01	L	L
**544610**	A (61.54)	G (70.00)	atoS	CDS	3E-03	1E-03	3.59	1.56	8.85	2.92	0.44	0.45	0.01	D	G
**859741**	A (60.00)	G (70.00)	NA	inter	3E-03	3E-03	3.59	1.56	8.85	2.92	0.44	0.45	0.02	NA	NA
**169619**	A (60.32)	A (96.67)	CTA_0156	CDS	4E-03	1E-04	0.05	0.00	0.25	-2.89	1.03	0.32	0.05	H	H
**170090**	G (60.32)	G (96.67)	CTA_0156	CDS	4E-03	1E-04	19.86	4.04	359.59	2.89	1.03	0.32	0.06	R	R
**544142**	G (61.24)	A (70.00)	CTA_0510	CDS	4E-03	1E-04	0.28	0.11	0.65	-2.89	0.44	0.45	0.03	E	E
**783158**	G (59.68)	G (96.67)	NA	inter	4E-03	1E-04	19.74	4.01	357.39	2.89	1.03	0.32	0.07	NA	NA
**413069**	G (60.77)	G (96.67)	bioY	CDS	4E-03	3E-03	19.46	3.96	352.15	2.87	1.03	0.33	0.01	A	A
**322838**	C (60.16)	C (90.00)	trmU	CDS	4E-03	1E-04	6.20	2.05	26.94	2.87	0.64	0.34	0.04	T	T
**296666**	T (60.94)	C (70.00)	CTA_0285	CDS	4E-03	1E-04	0.29	0.12	0.66	-2.86	0.44	0.45	0.03	V	V
**297482**	G (60.94)	A (70.00)	msbA	CDS	4E-03	1E-04	0.29	0.12	0.66	-2.86	0.44	0.45	0.05	C	C
**166931**	C (61.24)	C (96.67)	CTA_0156	CDS	4E-03	1E-04	0.05	0.00	0.26	-2.85	1.03	0.32	0.02	A	A
**179363**	T (61.42)	T (96.67)	CTA_0163	CDS	4E-03	1E-04	18.95	3.85	343.00	2.85	1.03	0.32	0.03	K	K
**978062**	C (61.42)	C (96.67)	nrdA	CDS	4E-03	1E-04	18.95	3.85	343.00	2.85	1.03	0.32	0.04	L	L
**153017**	C (61.60)	C (96.67)	CTA_0142	CDS	5E-03	1E-04	0.05	0.00	0.26	-2.84	1.03	0.31	0.08	A	A
**783136**	T (60.66)	T (90.00)	NA	inter	5E-03	1E-04	6.08	2.00	26.48	2.84	0.64	0.32	0.08	NA	NA
**68334**	T (61.72)	T (96.67)	CTA_0062	CDS	5E-03	1E-04	18.70	3.80	338.46	2.83	1.03	0.32	0.04	M	M

**189084**	A (61.48)	A (93.33)	NA	inter	5E-03	1E-04	0.12	0.02	0.42	-2.83	0.76	0.30	0.09	NA	NA
**96018**	C (60.77)	C (90.00)	CTA_0087	CDS	5E-03	5E-03	0.17	0.04	0.50	-2.83	0.63	0.34	0.03	H	H
**543548**	T (60.63)	C (70.00)	CTA_0508	CDS	5E-03	1E-04	0.29	0.12	0.67	-2.83	0.44	0.45	0.06	F	S
**170209**	T (61.90)	T (96.67)	CTA_0156	CDS	5E-03	1E-04	18.56	3.77	336.07	2.83	1.03	0.31	0.05	L	L
**170705**	A (61.90)	A (96.67)	CTA_0156	CDS	5E-03	1E-04	0.05	0.00	0.27	-2.83	1.03	0.31	0.06	K	K
**778572**	A (51.61)	G (79.31)	rrf	CDS	5E-03	1E-04	4.03	1.62	11.53	2.83	0.49	0.43	0.09	S	S
**168507**	A (62.20)	A (96.67)	CTA_0156	CDS	5E-03	1E-04	0.05	0.00	0.27	-2.81	1.03	0.31	0.05	E	E
**162304**	C (62.50)	C (96.67)	NA	inter	5E-03	1E-04	0.06	0.00	0.27	-2.80	1.03	0.31	0.05	NA	NA
**163924**	C (62.79)	C (96.67)	CTA_0154	CDS	5E-03	1E-04	0.06	0.00	0.28	-2.79	1.03	0.31	0.02	A	A
**157406**	T (63.08)	T (96.67)	oppA_1	CDS	5E-03	5E-03	17.62	3.58	318.95	2.78	1.03	0.31	0.01	S	S
**160656**	C (63.08)	C (96.67)	CTA_0152	CDS	5E-03	6E-03	0.06	0.00	0.28	-2.78	1.03	0.31	0.01	T	T
**162727**	T (63.08)	T (96.67)	CTA_0154	CDS	5E-03	4E-03	17.62	3.58	318.95	2.78	1.03	0.31	0.01	T	T
**162924**	C (63.08)	C (96.67)	CTA_0154	CDS	5E-03	4E-03	17.62	3.58	318.95	2.78	1.03	0.31	0.02	S	S
**163798**	C (63.08)	C (96.67)	CTA_0154	CDS	5E-03	4E-03	0.06	0.00	0.28	-2.78	1.03	0.31	0.01	G	G
**163870**	T (63.08)	T (96.67)	CTA_0154	CDS	5E-03	4E-03	17.62	3.58	318.95	2.78	1.03	0.31	0.01	Y	Y
**164065**	G (63.08)	G (96.67)	CTA_0154	CDS	5E-03	5E-03	17.62	3.58	318.95	2.78	1.03	0.31	0.03	V	V
**510718**	C (59.23)	T (70.00)	rpsL	CDS	6E-03	5E-03	3.37	1.47	8.28	2.77	0.44	0.46	0.02	E	E
**780057**	C (60.00)	C (92.31)	tsf	CDS	6E-03	1E-04	8.05	2.25	51.55	2.75	0.76	0.32	0.10	R	R
**776971**	A (58.27)	A (86.67)	karG	CDS	6E-03	1E-04	0.21	0.06	0.59	-2.73	0.57	0.36	0.05	V	V
**776973**	C (58.27)	C (86.67)	karG	CDS	6E-03	1E-04	0.21	0.06	0.59	-2.73	0.57	0.36	0.05	V	V
**776974**	T (58.27)	T (86.67)	karG	CDS	6E-03	1E-04	4.69	1.70	16.63	2.73	0.57	0.36	0.05	L	L
**969583**	T (58.73)	C (70.00)	sucD	CDS	7E-03	1E-04	0.30	0.12	0.70	-2.72	0.44	0.46	0.04	L	P
**443826**	C (64.84)	C (96.67)	CTA_0422	CDS	7E-03	1E-04	0.06	0.00	0.30	-2.70	1.03	0.29	0.02	T	T
**94278**	T (65.62)	T (93.33)	NA	inter	7E-03	1E-04	7.60	2.15	48.48	2.69	0.76	0.29	0.05	NA	NA
**777101**	C (59.23)	C (88.89)	karG	CDS	7E-03	1E-04	0.18	0.04	0.55	-2.68	0.64	0.35	0.05	C	C
**366448**	G (60.77)	A (66.67)	CTA_0348	CDS	9E-03	5E-03	0.32	0.14	0.74	-2.63	0.43	0.45	0.02	L	L
**410851**	A (60.77)	A (86.67)	yyaL	CDS	9E-03	9E-03	0.23	0.06	0.63	-2.60	0.57	0.35	0.02	A	A

**780167**	G (63.49)	G (96.00)	tsf	CDS	1E-02	1E-04	14.34	2.88	260.56	2.57	1.04	0.30	0.08	V	V
**429809**	A (60.47)	A (86.67)	arcD_2	CDS	1E-02	1E-04	0.23	0.07	0.64	-2.56	0.57	0.34	0.04	K	K
**44611**	C (60.63)	T (66.67)	CTA_0043	CDS	1E-02	1E-04	2.96	1.30	7.10	2.53	0.43	0.45	0.04	A	V
**777111**	A (58.91)	A (85.71)	karG	CDS	1E-02	1E-04	0.24	0.07	0.66	-2.53	0.57	0.36	0.04	L	L
**533906**	T (74.62)	C (50.00)	CTA_0498	CDS	1E-02	9E-03	0.35	0.15	0.80	-2.51	0.42	0.31	0.01	L	P
**162020**	G (62.20)	G (86.67)	CTA_0153	CDS	1E-02	1E-04	4.11	1.49	14.55	2.49	0.57	0.33	0.04	G	G
**162023**	A (62.20)	A (86.67)	CTA_0153	CDS	1E-02	1E-04	0.24	0.07	0.67	-2.49	0.57	0.33	0.03	G	G
**781610**	T (56.76)	T (94.44)	NA	inter	1E-02	1E-04	13.38	2.60	245.28	2.48	1.05	0.31	0.22	NA	NA
**983698**	G (62.79)	G (86.67)	murB	CDS	1E-02	1E-04	4.00	1.45	14.17	2.44	0.57	0.33	0.02	H	H
**971312**	T (70.40)	T (96.67)	NA	inter	2E-02	1E-04	0.08	0.00	0.41	-2.41	1.04	0.24	0.06	NA	NA
**366656**	A (60.16)	A (83.33)	CTA_0348	CDS	2E-02	1E-04	0.30	0.10	0.78	-2.30	0.52	0.35	0.04	I	I
**437629**	A (67.44)	A (90.00)	NA	inter	2E-02	1E-04	0.23	0.05	0.70	-2.30	0.64	0.28	0.02	NA	NA
**295635**	G (61.24)	A (63.33)	CTA_0284	CDS	2E-02	1E-04	0.38	0.16	0.86	-2.30	0.42	0.44	0.03	R	K
**162017**	A (62.20)	A (83.33)	CTA_0153	CDS	3E-02	1E-04	0.32	0.10	0.82	-2.20	0.52	0.34	0.04	V	V
**782876**	A (62.50)	A (83.33)	NA	inter	3E-02	1E-04	0.32	0.10	0.83	-2.17	0.52	0.34	0.03	NA	NA
**168430**	G (62.79)	G (83.33)	CTA_0156	CDS	3E-02	1E-04	3.08	1.19	9.60	2.15	0.52	0.34	0.04	V	V
**180271**	A (62.99)	A (93.75)	NA	inter	3E-02	1E-04	0.11	0.01	0.57	-2.11	1.05	0.31	0.13	NA	NA
**779939**	G (59.09)	G (87.50)	tsf	CDS	4E-02	1E-04	5.08	1.33	33.39	2.08	0.78	0.30	0.23	G	G
**656336**	A (71.09)	A (90.00)	CTA_0630	CDS	4E-02	1E-04	0.27	0.06	0.84	-2.02	0.64	0.25	0.03	L	L
**46460**	A (60.77)	G (60.00)	CTA_0045	CDS	5E-02	3E-02	2.24	1.00	5.15	1.94	0.41	0.44	0.02	V	V
**95527**	C (60.77)	T (60.00)	CTA_0087	CDS	5E-02	4E-02	2.24	1.00	5.15	1.94	0.41	0.44	0.01	S	L
**780308**	A (61.42)	A (81.48)	tsf	CDS	5E-02	1E-04	0.36	0.11	0.95	-1.93	0.53	0.34	0.07	H	H
**413567**	A (60.47)	G (60.00)	CTA_0391	CDS	6E-02	1E-04	2.21	0.99	5.08	1.91	0.41	0.44	0.04	V	A
**780493**	C (57.98)	A (62.07)	rpsB	CDS	6E-02	1E-04	0.45	0.19	1.02	-1.89	0.43	0.43	0.11	V	V
**162003**	C (62.20)	C (80.00)	CTA_0153	CDS	6E-02	1E-04	0.40	0.14	0.98	-1.88	0.49	0.34	0.05	L	L
**790520**	A (51.16)	A (70.00)	CTA_0747	CDS	6E-02	1E-04	0.44	0.18	1.01	-1.87	0.44	0.45	0.03	G	G
**100191**	A (58.91)	G (60.00)	NA	inter	7E-02	1E-04	2.13	0.96	4.91	1.83	0.41	0.45	0.04	NA	NA

**1027490**	G (58.91)	T (60.00)	CTA_0948	CDS	7E-02	1E-04	2.13	0.96	4.91	1.83	0.41	0.45	0.01	P	Q
**161950**	C (61.90)	C (79.31)	CTA_0153	CDS	7E-02	1E-04	2.45	0.98	7.03	1.82	0.49	0.34	0.06	A	A
**777183**	T (58.59)	C (60.00)	karG	CDS	7E-02	1E-04	0.47	0.21	1.06	-1.80	0.41	0.45	0.04	I	V
**780284**	A (61.42)	A (80.00)	tsf	CDS	8E-02	1E-04	0.40	0.13	1.05	-1.74	0.53	0.34	0.08	A	A
