# Supplementary material for: Population-based analysis of ocular Chlamydia trachomatis in trachoma-endemic West African communities identifies genomic markers of disease severity
Source: Genome Med. 2018 Feb 26;10:15. doi: 10.1186/s13073-018-0521-x (PMC5828069; doi:10.1186/s13073-018-0521-x)
Supplement: Supplementary file 10 — Figure S10. SNPs across the Chlamydia trachomatis genome associated with disease severity using permutation-based genome-wide association analysis. (PDF 158 kb) [file 13073_2018_521_MOESM10_ESM.pdf]

Figure S10. SNPs across the *Chlamydia trachomatis* genome associated with disease severity using permutation-based genome-wide association analysis

Disease severity-associated SNPs (*p-value < 0.1*). Disease severity is defined by a composite *in vivo* conjunctival phenotype derived using principal component analysis using ocular *Ct* load and conjunctival inflammatory (P) score (using the modified FPC (Follicles, Papillary Hypertrophy, Conjunctival Scarring) grading system [39]. ‘Reference Allele’ indicates the reference allele on *Ct A/HAR-13* (GenBank Accession Number NC_007429). ‘CDS/NCR’ identifies whether the SNP was in a coding or non-coding region. ‘P*’=permuted p-value after 100,024 simulations indicating genome wide significance at *p*<0.05*. ‘T’ is the t statistic; SE(T) is the Standard Error of the t statistic. OR is the Odds Ratio (derived from the t statistic). 95% C.I.=95% confidence interval of the OR. ‘MAF’ is the minor allele frequency. ‘N Calls at Locus’ is the proportion of isolates which had no base called.

SNP POSITION	REFERENCE	ALTERNATIVE	GENE NAME	CDS/NCR	STRAND	P*	P-VALUE	T	SE(T)	OR	95% C.I.		MAF	N CALLS AT LOCUS
1028728	C	T	pmpE	CDS	-	0.013	0.011	-2.550	0.555	0.078	0.026	0.232	0.310	7.042
875804	C	T	alaS	CDS	-	0.024	0.022	-2.298	0.530	0.100	0.036	0.284	0.310	4.225
939488	G	A	glgA	CDS	-	0.026	0.023	-2.273	0.491	0.103	0.039	0.270	0.479	4.225
285610	G	A	CTA_0273	CDS	-	0.027	0.034	-2.123	0.526	0.120	0.043	0.336	0.310	4.225
32779	G	A	trmD	CDS	+	0.032	0.031	-2.160	0.525	0.115	0.041	0.323	0.310	2.817
465330	C	G	yjfH	CDS	-	0.037	0.042	-2.032	0.519	0.131	0.047	0.362	0.310	1.408
787841	A	G	NA	inter	NA	0.038	0.038	-2.074	0.524	0.126	0.045	0.351	0.310	4.225
827184	A	G	CTA_0774	CDS	+	0.041	0.043	-2.020	0.516	0.133	0.048	0.365	0.310	1.408
22049	G	T	ileS	CDS	+	0.057	0.050	-1.962	0.505	0.141	0.052	0.378	0.324	4.225
152011	G	A	NA	inter	NA	0.058	0.050	-1.964	0.505	0.140	0.052	0.377	0.324	4.225
710787	A	C	CTA_0675	CDS	-	0.060	0.052	-1.941	0.517	0.144	0.052	0.396	0.310	4.225
19085	T	C	NA	inter	NA	0.061	0.060	-1.882	0.530	0.152	0.054	0.430	0.296	5.634
388175	G	A	CTA_0368	CDS	-	0.061	0.059	-1.889	0.524	0.151	0.054	0.422	0.296	1.408
696782	A	T	rpoD	CDS	-	0.064	0.062	-1.864	0.511	0.155	0.057	0.422	0.310	1.408
286636	C	T	lgt	CDS	-	0.065	0.061	-1.876	0.511	0.153	0.056	0.417	0.310	0.000
930453	C	T	mutS	CDS	-	0.067	0.061	-1.876	0.511	0.153	0.056	0.417	0.310	0.000
465525	C	T	CTA_0439	CDS	-	0.067	0.062	-1.865	0.472	0.155	0.061	0.391	0.493	1.408
60858	G	A	CTA_0057	CDS	-	0.068	0.070	-1.813	0.512	0.163	0.060	0.445	0.310	1.408
835039	G	A	CTA_0782	CDS	-	0.070	0.061	-1.876	0.511	0.153	0.056	0.417	0.310	0.000
19005	A	G	NA	inter	NA	0.071	0.071	-1.807	0.525	0.164	0.059	0.459	0.296	2.817

4554	A	G	gatB	CDS	+	0.071	0.070	-1.813	0.512	0.163	0.060	0.445	0.310	1.408
303590	C	A	murE	CDS	-	0.072	0.061	-1.876	0.511	0.153	0.056	0.417	0.310	0.000
215130	C	T	gyrA_1	CDS	-	0.072	0.062	-1.864	0.511	0.155	0.057	0.422	0.310	1.408
806382	C	T	CTA_0761	CDS	+	0.073	0.058	-1.896	0.530	0.150	0.053	0.424	0.296	4.225
778783	G	A	rrf	CDS	-	0.077	0.075	-1.780	0.502	0.169	0.063	0.451	0.324	2.817
136812	G	A	incF	CDS	+	0.079	0.075	-1.780	0.502	0.169	0.063	0.451	0.324	2.817
169573	G	A	CTA_0156	CDS	+	0.082	0.077	-1.771	0.523	0.170	0.061	0.474	0.310	9.859
956953	C	T	pmpD	CDS	+	0.082	0.072	-1.800	0.523	0.165	0.059	0.461	0.296	2.817
44990	A	G	ruvB	CDS	+	0.087	0.086	-1.718	0.493	0.179	0.068	0.472	0.338	2.817
62140	G	T	sucA	CDS	+	0.091	0.078	-1.760	0.502	0.172	0.064	0.461	0.324	5.634
542521	G	A	CTA_0507	CDS	-	0.092	0.090	-1.696	0.494	0.183	0.070	0.483	0.338	2.817
181019	C	A	CTA_0164	CDS	-	0.095	0.096	-1.666	0.494	0.189	0.072	0.498	0.338	4.225
151156	C	G	CTA_0140	CDS	-	0.096	0.077	1.770	0.502	5.871	2.195	15.703	0.324	4.225
270020	T	C	NA	inter	NA	0.105	0.115	-1.576	0.506	0.207	0.077	0.558	0.310	5.634
176577	C	T	CTA_0162	CDS	-	0.106	0.090	-1.696	0.494	0.183	0.070	0.483	0.338	2.817
736806	G	C	ygeD	CDS	+	0.107	0.100	-1.642	0.513	0.194	0.071	0.529	0.310	9.859
756138	G	A	CTA_0714	CDS	+	0.107	0.106	-1.615	0.477	0.199	0.078	0.506	0.465	4.225
918317	G	A	CTA_0850	CDS	+	0.109	0.100	-1.647	0.496	0.193	0.073	0.510	0.338	2.817
151956	T	C	NA	inter	NA	0.110	0.091	-1.690	0.503	0.185	0.069	0.495	0.324	5.634
428879	G	A	arcD_3	CDS	+	0.111	0.108	-1.606	0.483	0.201	0.078	0.517	0.451	7.042
993154	T	C	ftsH	CDS	+	0.111	0.105	-1.622	0.498	0.197	0.074	0.524	0.324	2.817
862127	G	C	NA	inter	NA	0.112	0.104	-1.624	0.516	0.197	0.072	0.543	0.310	4.225
539885	T	G	yaeI	CDS	+	0.112	0.110	-1.597	0.496	0.203	0.077	0.535	0.338	4.225
722679	T	C	CTA_0686	CDS	-	0.116	0.127	-1.527	0.500	0.217	0.082	0.579	0.324	4.225
3653	G	T	gatA	CDS	+	0.117	0.112	-1.588	0.507	0.204	0.076	0.552	0.310	5.634
180907	G	A	CTA_0164	CDS	-	0.119	0.117	1.569	0.489	4.801	1.840	12.531	0.338	1.408
540002	G	A	yaeI	CDS	+	0.120	0.117	1.569	0.489	4.801	1.840	12.531	0.338	1.408
2584	A	G	gatC	CDS	+	0.122	0.124	-1.538	0.498	0.215	0.081	0.570	0.324	4.225
349027	C	T	CTA_0331	CDS	-	0.122	0.119	-1.559	0.490	0.210	0.081	0.550	0.338	2.817
251666	A	G	yqfU	CDS	-	0.123	0.116	-1.573	0.497	0.207	0.078	0.549	0.338	5.634
180033	A	G	CTA_0163	CDS	-	0.123	0.130	1.515	0.491	4.551	1.737	11.925	0.338	1.408
27553	G	A	CTA_0023	CDS	-	0.125	0.113	-1.585	0.489	0.205	0.079	0.535	0.338	2.817
458680	C	T	CTA_0433	CDS	+	0.125	0.097	-1.659	0.518	0.190	0.069	0.525	0.296	1.408
769145	T	C	CTA_0727	CDS	+	0.126	0.117	-1.569	0.489	0.208	0.080	0.544	0.338	1.408

599664	G	A	CTA_0578	CDS	-	0.128	0.143	-1.463	0.484	0.231	0.090	0.597	0.352	1.408
107371	A	G	NA	inter	NA	0.128	0.132	-1.508	0.490	0.221	0.085	0.579	0.338	2.817
167953	G	A	CTA_0156	CDS	+	0.129	0.114	-1.583	0.489	0.205	0.079	0.536	0.338	2.817
382260	G	A	uvrA	CDS	+	0.130	0.117	-1.569	0.489	0.208	0.080	0.544	0.338	1.408
646594	C	G	gspD_1	CDS	-	0.136	0.130	-1.515	0.491	0.220	0.084	0.576	0.338	1.408
381555	G	C	uvrA	CDS	+	0.139	0.130	-1.515	0.491	0.220	0.084	0.576	0.338	1.408
20824	G	A	CTA_0019	CDS	-	0.141	0.127	-1.525	0.507	0.218	0.081	0.588	0.310	7.042
174222	G	A	CTA_0160	CDS	+	0.142	0.133	-1.504	0.497	0.222	0.084	0.589	0.338	5.634
562019	C	A	CTA_0529	CDS	-	0.143	0.141	-1.472	0.522	0.230	0.083	0.639	0.296	8.451
53545	C	T	CTA_0051	CDS	+	0.144	0.143	-1.463	0.484	0.231	0.090	0.597	0.352	1.408
179438	A	G	CTA_0163	CDS	-	0.144	0.117	-1.569	0.489	0.208	0.080	0.544	0.338	1.408
596140	C	T	rplV	CDS	-	0.148	0.132	-1.507	0.492	0.222	0.084	0.581	0.338	2.817
222278	C	T	CTA_0213	CDS	-	0.154	0.143	-1.463	0.484	0.231	0.090	0.597	0.352	1.408
152630	G	A	CTA_0141	CDS	+	0.154	0.146	-1.455	0.499	0.233	0.088	0.621	0.324	5.634
641223	G	A	CTA_0618	CDS	-	0.156	0.167	-1.383	0.504	0.251	0.093	0.674	0.324	5.634
597552	C	T	rplW	CDS	-	0.160	0.166	-1.386	0.488	0.250	0.096	0.650	0.338	1.408
443826	C	T	CTA_0422	CDS	+	0.163	0.152	-1.433	0.502	0.239	0.089	0.638	0.324	5.634
458077	A	G	vacB	CDS	+	0.164	0.166	-1.386	0.488	0.250	0.096	0.650	0.338	1.408
611163	T	C	aspS	CDS	-	0.170	0.181	-1.338	0.480	0.262	0.102	0.671	0.352	0.000
580167	G	A	gidA	CDS	+	0.170	0.181	-1.338	0.480	0.262	0.102	0.671	0.352	0.000
647303	C	T	CTA_0623	CDS	-	0.170	0.181	-1.338	0.480	0.262	0.102	0.671	0.352	0.000
873803	C	T	mfd	CDS	-	0.176	0.181	-1.338	0.480	0.262	0.102	0.671	0.352	0.000
5079	C	A	gatB	CDS	+	0.178	0.181	-1.338	0.480	0.262	0.102	0.671	0.352	0.000
312933	G	A	nqrC	CDS	+	0.179	0.181	-1.338	0.480	0.262	0.102	0.671	0.352	0.000
1022981	A	G	CTA_0945	CDS	+	0.180	0.181	-1.338	0.480	0.262	0.102	0.671	0.352	0.000
894	G	A	CTA_0001	CDS	+	0.181	0.181	-1.338	0.480	0.262	0.102	0.671	0.352	0.000
152319	A	G	NA	inter	NA	0.181	0.181	-1.338	0.480	0.262	0.102	0.671	0.352	0.000
828422	T	C	ompB	CDS	-	0.182	0.165	-1.388	0.494	0.250	0.095	0.657	0.324	2.817
169913	G	T	CTA_0156	CDS	+	0.182	0.194	-1.300	0.519	0.273	0.098	0.754	0.324	15.493
621170	C	T	NA	inter	NA	0.183	0.181	-1.338	0.480	0.262	0.102	0.671	0.352	0.000
804958	G	A	psdD	CDS	+	0.184	0.183	-1.332	0.480	0.264	0.103	0.677	0.352	1.408
930869	G	A	mutS	CDS	-	0.186	0.181	-1.338	0.480	0.262	0.102	0.671	0.352	0.000
180479	T	C	NA	inter	NA	0.188	0.181	-1.338	0.480	0.262	0.102	0.671	0.352	0.000
87637	G	T	yaeL	CDS	+	0.189	0.183	-1.332	0.480	0.264	0.103	0.677	0.352	1.408

922798	C	A	CTA_0853	CDS	+	0.189	0.181	-1.338	0.480	0.262	0.102	0.671	0.352	0.000	
451127	C	T	proS	CDS	+	0.189	0.181	-1.338	0.480	0.262	0.102	0.671	0.352	0.000	
1037066	A	T	pmpG	CDS	+	0.190	0.181	-1.338	0.480	0.262	0.102	0.671	0.352	0.000	
578122	G	A	dnaB_1	CDS	+	0.190	0.181	-1.338	0.480	0.262	0.102	0.671	0.352	0.000	
642364	C	T	gspF	CDS	-	0.190	0.181	-1.338	0.480	0.262	0.102	0.671	0.352	0.000	
390866	C	T	pdhA_2	CDS	-	0.191	0.181	-1.338	0.480	0.262	0.102	0.671	0.352	0.000	
595805	C	T	rpsC	CDS	-	0.193	0.181	-1.338	0.480	0.262	0.102	0.671	0.352	0.000	
165708	G	A	dnlJ	CDS	+	0.198	0.181	-1.338	0.480	0.262	0.102	0.671	0.352	0.000	
1010926	A	G	CTA_0934	CDS	-	0.198	0.181	-1.338	0.480	0.262	0.102	0.671	0.352	0.000	
226817	G	A	oppB_1	CDS	+	0.200	0.182	-1.336	0.494	0.263	0.100	0.693	0.338	5.634	
472850	G	A	lpxB	CDS	+	0.201	0.181	-1.338	0.480	0.262	0.102	0.671	0.352	0.000	
734086	G	T	recC	CDS	-	0.201	0.181	-1.338	0.480	0.262	0.102	0.671	0.352	0.000	
180328	G	A	NA	inter	NA	0.202	0.189	-1.314	0.502	0.269	0.100	0.719	0.324	7.042	
748131	C	T	recA	CDS	+	0.204	0.217	-1.236	0.482	0.291	0.113	0.747	0.352	1.408	
176292	A	G	NA	inter	NA	0.205	0.218	-1.233	0.482	0.292	0.113	0.749	0.352	1.408	
1035739	G	A	pmpG	CDS	+	0.206	0.181	-1.338	0.480	0.262	0.102	0.671	0.352	0.000	
656197	G	A	CTA_0630	CDS	-	0.207	0.183	-1.332	0.480	0.264	0.103	0.677	0.352	1.408	
30512	G	A	CTA_0026	CDS	+	0.210	0.211	-1.252	0.481	0.286	0.111	0.734	0.352	1.408	
149025	C	T	CTA_0138	CDS	-	0.216	0.211	-1.252	0.481	0.286	0.111	0.734	0.352	1.408	
168530	A	G	CTA_0156	CDS	+	0.218	0.211	-1.252	0.481	0.286	0.111	0.734	0.352	1.408	
100125	G	A	CTA_0090	CDS	-	0.222	0.220	-1.226	0.482	0.293	0.114	0.754	0.352	1.408	
26497	A	G	lepB	CDS	-	0.228	0.253	-1.142	0.483	0.319	0.124	0.823	0.352	2.817	
486369	A	C	pmpC	CDS	+	0.228	0.217	-1.236	0.482	0.291	0.113	0.747	0.352	1.408	
258291	C	A	CTA_0251	CDS	-	0.230	0.189	-1.315	0.502	0.269	0.100	0.719	0.324	8.451	
252714	A	G	NA	inter	NA	0.238	0.220	-1.227	0.482	0.293	0.114	0.754	0.352	2.817	
345498	T	C	atpD	CDS	-	0.238	0.233	1.193	0.499	3.295	1.240	8.756	0.324	4.225	
76491	A	G	lepA	CDS	-	0.238	0.249	-1.152	0.485	0.316	0.122	0.818	0.338	2.817	
644112	G	T	gspE	CDS	-	0.245	0.217	-1.236	0.482	0.291	0.113	0.747	0.352	1.408	
29521	A	G	prfA	CDS	+	0.247	0.217	-1.236	0.482	0.291	0.113	0.747	0.352	1.408	
197795	G	A	CTA_0187	CDS	-	0.252	0.260	-1.126	0.484	0.324	0.126	0.837	0.352	2.817	
412251	C	T	CTA_0389	CDS	+	0.260	0.261	-1.123	0.484	0.325	0.126	0.840	0.352	4.225	
534641	G	A	CTA_0498	CDS	+	0.261	0.254	-1.140	0.491	0.320	0.122	0.837	0.352	7.042	
265183	C	T	CTA_0256	CDS	+	0.270	0.239	-1.178	0.490	0.308	0.118	0.805	0.338	4.225	
556127	G	A	oppC_2	CDS	-	0.282	0.261	-1.124	0.510	0.325	0.120	0.883	0.296	8.451	

698839	T	C	CTA_0669	CDS	-	0.287	0.285	-1.068	0.486	0.344	0.133	0.891	0.338	2.817
1015112	C	G	ispH	CDS	+	0.290	0.292	-1.053	0.486	0.349	0.134	0.905	0.338	2.817
1031113	G	A	pmpE	CDS	-	0.298	0.287	-1.064	0.501	0.345	0.129	0.921	0.324	5.634
295401	C	T	CTA_0284	CDS	+	0.300	0.285	-1.068	0.486	0.344	0.133	0.891	0.338	2.817
50284	C	T	NA	inter	NA	0.303	0.293	-1.051	0.494	0.350	0.133	0.920	0.338	8.451
534599	A	G	CTA_0498	CDS	+	0.314	0.299	1.038	0.487	2.824	1.087	7.336	0.338	4.225
144878	G	T	NA	inter	NA	0.319	0.297	-1.044	0.487	0.352	0.136	0.914	0.338	2.817
