# Supplementary material for: Population-based analysis of ocular Chlamydia trachomatis in trachoma-endemic West African communities identifies genomic markers of disease severity
Source: Genome Med. 2018 Feb 26;10:15. doi: 10.1186/s13073-018-0521-x (PMC5828069; doi:10.1186/s13073-018-0521-x)
Supplement: Supplementary file 11 — Figure S11. Summary of published studies supporting the key ocular localization and disease severity-associated SNPs [106–114]. (PDF 105 kb) [file 13073_2018_521_MOESM11_ESM.pdf]

Figure S11. Summary of published studies supporting the key ocular localization and disease severity-associated SNPs

NAME	ASSOCIATION	EXPRESSION STAGE*	LOCALIZATION	POLYMORPHISM	FUNCTION
alaS	Disease severity	Mid to late	Unknown	NA	tRNA ligase [99,100]
ChlaDub1	Ocular localization	Mid to late	Host cytosol [106]	Within/between clades [8,93]	Prevents NFκB activation [106,107]
CTA0273	Disease severity	Late	Unknown	NA	Membrane insertase [101,102]
CTA0744	Disease severity	Mid to late	Unknown	NA	Unknown
EEA1	Ocular localization	Early	Inclusion membrane [73]	Within/between clades [8,91]	Inclusion trafficking [73]
glgA	Disease severity	Mid to late	Host cytosol [108,109]	NA	Glycogen biosynthesis
pmpD	Ocular localization	Late	EB-surface [110,111]	Within/between clades [20,112]	Adhesion and transport [15,110,113]
pmpE	Disease severity	Mid to late	EB-surface [114]	Within/between clades [20,112]	Adhesion and transport [15,110,113]
tarP	Ocular localization	Mid to late	EB-surface [94]	Within/between clades [8,91]	Cell entry [94]
trmD	Disease severity	Early	Unknown	NA	tRNA methyl-transferase [73]

yjfH (rlmB)	Disease severity	Early	Unknown	NA	Ribosome assembly [103]

*Early = 1-3 hours post infection [HPI]; Mid to late = 16-24 HPI; Late = 24-40 HPI as defined by Belland *et al*. [73].
