# Supplementary material for: Population-based analysis of ocular Chlamydia trachomatis in trachoma-endemic West African communities identifies genomic markers of disease severity
Source: Genome Med. 2018 Feb 26;10:15. doi: 10.1186/s13073-018-0521-x (PMC5828069; doi:10.1186/s13073-018-0521-x)
Supplement: Supplementary file 12 — Figure S12. European Nucleotide Archive (ENA) (European Bioinformatics Institute (EBI)) accession numbers relating to C. trachomatis sequence data analysed in this study. (PDF 75 kb) [file 13073_2018_521_MOESM12_ESM.pdf]

Figure S12. European Nucleotide Archive (ENA) (European Bioinformatics Institute (EBI)) accession numbers relating to *C. trachomatis* sequence data analysed in this study.

Study_accession	Sample_accession	Experiment_accession	Run_accession	sample_alias	Sequencing_ID	File_name
PRJEB12542	ERS1047004	ERX1303411	ERR1231472	Ctr1	11152_3_10	TcSample_14344
PRJEB12542	ERS1047005	ERX1303412	ERR1231473	Ctr2	11152_3_11	TcSample_17347
PRJEB12542	ERS1047006	ERX1303413	ERR1231474	Ctr3	11152_3_1	TcSample_4422
PRJEB12542	ERS1047007	ERX1303414	ERR1231475	Ctr4	11152_3_12	TcSample_11231
PRJEB12542	ERS1047008	ERX1303415	ERR1231476	Ctr5	11152_3_13	TcSample_15631
PRJEB12542	ERS1047009	ERX1303416	ERR1231477	Ctr6	11152_3_14	TcSample_6105
PRJEB12542	ERS1047010	ERX1303417	ERR1231478	Ctr7	11152_3_15	TcSample_12628
PRJEB12542	ERS1047011	ERX1303418	ERR1231479	Ctr8	11152_3_16	TcSample_7524
PRJEB12542	ERS1047012	ERX1303419	ERR1231480	Ctr9	11152_3_17	TcSample_5016
PRJEB12542	ERS1047013	ERX1303420	ERR1231481	Ctr10	11152_3_18	TcSample_1485
PRJEB12542	ERS1047014	ERX1303421	ERR1231482	Ctr11	11152_3_19	TcSample_15554
PRJEB12542	ERS1047015	ERX1303422	ERR1231483	Ctr12	11152_3_20	TcSample_6094
PRJEB12542	ERS1047016	ERX1303423	ERR1231484	Ctr13	11152_3_21	TcSample_3168
PRJEB12542	ERS1047017	ERX1303424	ERR1231485	Ctr14	11152_3_22	TcSample_5082
PRJEB12542	ERS1047018	ERX1303425	ERR1231486	Ctr15	11152_3_23	TcSample_12969
PRJEB12542	ERS1047019	ERX1303426	ERR1231487	Ctr16	11152_3_25	TcSample_8140
PRJEB12542	ERS1047020	ERX1303427	ERR1231488	Ctr17	11152_3_26	TcSample_6083
PRJEB12542	ERS1047021	ERX1303428	ERR1231489	Ctr18	11152_3_27	TcSample_16621
PRJEB12542	ERS1047022	ERX1303429	ERR1231490	Ctr19	11152_3_28	TcSample_16852
PRJEB12542	ERS1047023	ERX1303430	ERR1231491	Ctr20	11152_3_29	TcSample_16588
PRJEB12542	ERS1047024	ERX1303431	ERR1231492	Ctr21	11152_3_30	TcSample_4180
PRJEB12542	ERS1047025	ERX1303432	ERR1231493	Ctr22	11152_3_31	TcSample_7612
PRJEB12542	ERS1047026	ERX1303433	ERR1231494	Ctr23	11152_3_3	TcSample_6985
PRJEB12542	ERS1047027	ERX1303434	ERR1231495	Ctr24	11152_3_32	TcSample_4411
PRJEB12542	ERS1047028	ERX1303435	ERR1231496	Ctr25	11152_3_33	TcSample_4257

PRJEB12542	ERS1047029	ERX1303436	ERR1231497	Ctr26	11152_3_34	TcSample_4400
PRJEB12542	ERS1047030	ERX1303437	ERR1231498	Ctr27	11152_3_35	TcSample_15180
PRJEB12542	ERS1047031	ERX1303438	ERR1231499	Ctr28	11152_3_36	TcSample_13596
PRJEB12542	ERS1047032	ERX1303439	ERR1231500	Ctr29	11152_3_37	TcSample_1672
PRJEB12542	ERS1047033	ERX1303440	ERR1231501	Ctr30	11152_3_38	TcSample_5181
PRJEB12542	ERS1047034	ERX1303441	ERR1231502	Ctr31	11152_3_39	TcSample_15532
PRJEB12542	ERS1047035	ERX1303442	ERR1231503	Ctr32	11152_3_40	TcSample_8074
PRJEB12542	ERS1047036	ERX1303443	ERR1231504	Ctr33	11152_3_41	TcSample_16984
PRJEB12542	ERS1047037	ERX1303444	ERR1231505	Ctr34	11152_3_4	TcSample_1881
PRJEB12542	ERS1047038	ERX1303445	ERR1231506	Ctr35	11152_3_42	TcSample_10032
PRJEB12542	ERS1047039	ERX1303446	ERR1231507	Ctr36	11152_3_43	TcSample_8492
PRJEB12542	ERS1047040	ERX1303447	ERR1231508	Ctr37	11152_3_44	TcSample_13585
PRJEB12542	ERS1047041	ERX1303448	ERR1231509	Ctr38	11152_3_45	TcSample_4224
PRJEB12542	ERS1047042	ERX1303449	ERR1231510	Ctr39	11152_3_47	TcSample_2640
PRJEB12542	ERS1047043	ERX1303450	ERR1231511	Ctr40	11152_3_48	TcSample_7535
PRJEB12542	ERS1047044	ERX1303451	ERR1231512	Ctr41	11152_3_50	TcSample_7095
PRJEB12542	ERS1047045	ERX1303452	ERR1231513	Ctr42	11152_3_5	TcSample_6028
PRJEB12542	ERS1047046	ERX1303453	ERR1231514	Ctr43	11152_3_52	TcSample_10021
PRJEB12542	ERS1047047	ERX1303454	ERR1231515	Ctr44	11152_3_53	TcSample_15378
PRJEB12542	ERS1047048	ERX1303455	ERR1231516	Ctr45	11152_3_54	TcSample_12804
PRJEB12542	ERS1047049	ERX1303456	ERR1231517	Ctr46	11152_3_55	TcSample_12650
PRJEB12542	ERS1047050	ERX1303457	ERR1231518	Ctr47	11152_3_57	TcSample_8965
PRJEB12542	ERS1047051	ERX1303458	ERR1231519	Ctr48	11152_3_58	TcSample_5104
PRJEB12542	ERS1047052	ERX1303459	ERR1231520	Ctr49	11152_3_60	TcSample_16599
PRJEB12542	ERS1047053	ERX1303460	ERR1231521	Ctr50	11152_3_6	TcSample_4928
PRJEB12542	ERS1047054	ERX1303461	ERR1231522	Ctr51	11152_3_62	TcSample_7062
PRJEB12542	ERS1047055	ERX1303462	ERR1231523	Ctr52	11152_3_63	TcSample_8778
PRJEB12542	ERS1047056	ERX1303463	ERR1231524	Ctr53	11152_3_66	TcSample_1892
PRJEB12542	ERS1047057	ERX1303464	ERR1231525	Ctr54	11152_3_69	TcSample_11495

PRJEB12542	ERS1047058	ERX1303465	ERR1231526	Ctr55	11152_3_70	TcSample_10747
PRJEB12542	ERS1047059	ERX1303466	ERR1231527	Ctr56	11152_3_7	TcSample_13189
PRJEB12542	ERS1047060	ERX1303467	ERR1231528	Ctr57	11152_3_74	TcSample_15499
PRJEB12542	ERS1047061	ERX1303468	ERR1231529	Ctr58	11152_3_76	TcSample_726
PRJEB12542	ERS1047062	ERX1303469	ERR1231530	Ctr59	11152_3_77	TcSample_7579
PRJEB12542	ERS1047063	ERX1303470	ERR1231531	Ctr60	11152_3_78	TcSample_12089
PRJEB12542	ERS1047064	ERX1303471	ERR1231532	Ctr61	11152_3_8	TcSample_6996
PRJEB12542	ERS1047065	ERX1303472	ERR1231533	Ctr62	11152_3_88	TcSample_748
PRJEB12542	ERS1047066	ERX1303473	ERR1231534	Ctr63	11152_3_9	TcSample_10967
PRJEB12542	ERS1047067	ERX1303474	ERR1231535	Ctr64	11152_3_92	TcSample_1463
PRJEB12542	ERS1047068	ERX1303475	ERR1231536	Ctr65	11152_3_94	TcSample_1683
PRJEB12542	ERS1047069	ERX1303476	ERR1231537	Ctr66	13108_1_12	TcSample_22121
PRJEB12542	ERS1047070	ERX1303477	ERR1231538	Ctr67	13108_1_14	TcSample_24519
PRJEB12542	ERS1047071	ERX1303478	ERR1231539	Ctr68	13108_1_15	TcSample_6941
PRJEB12542	ERS1047072	ERX1303479	ERR1231540	Ctr69	13108_1_2	TcSample_27742
PRJEB12542	ERS1047073	ERX1303480	ERR1231541	Ctr70	13108_1_7	TcSample_25124
PRJEB12542	ERS1047074	ERX1303481	ERR1231542	Ctr71	13108_1_9	TcSample_22154
PRJEB12542	ERS1047075	ERX1303482	ERR1231543	Ctr72	8422_8_49	TcSample_2353
PRJEB12542	ERS1047076	ERX1303483	ERR1231544	Ctr73	8422_8_50	TcSample_2366
PRJEB12542	ERS1047077	ERX1303484	ERR1231545	Ctr74	9471_4_86	TcSample_12980
PRJEB12542	ERS1047078	ERX1303485	ERR1231546	Ctr75	9471_4_87	TcSample_15367
PRJEB12542	ERS1047079	ERX1303486	ERR1231547	Ctr76	9471_4_88	TcSample_15543
PRJEB12542	ERS1047080	ERX1303487	ERR1231548	Ctr77	9471_4_89	TcSample_1870
PRJEB12542	ERS1047081	ERX1303488	ERR1231549	Ctr78	9471_4_90	TcSample_2145
PRJEB12542	ERS1047082	ERX1303489	ERR1231550	Ctr79	9471_4_91	TcSample_4158
PRJEB12542	ERS1047083	ERX1303490	ERR1231551	Ctr80	9471_4_92	TcSample_4169
PRJEB12542	ERS1047084	ERX1303491	ERR1231552	Ctr81	9471_4_93	TcSample_7590
PRJEB12542	ERS1047085	ERX1303492	ERR1231553	Ctr82	9519_1_41	TcSample_117
PRJEB12542	ERS1047086	ERX1303493	ERR1231554	Ctr83	9519_1_44	TcSample_2405

PRJEB2214	ERS017900	ERX012723	ERR034213	A_MH363	5766_7_3	363
PRJEB2214	ERS017901	ERX012723	ERR034214	A_MH5291	5766_7_4	5291
PRJEB2214	ERS017902	ERX012723	ERR034215	A_MH7249	5766_7_5	7249
PRJEB2214	ERS177716	ERX151706	ERR175560	A_MH858	5965_2_1	858
PRJEB2214	ERS177725	ERX151715	ERR175569	A_MH18843	5965_2_10	18843
PRJEB2214	ERS177726	ERX151716	ERR175570	A_MH19657	5965_2_11	19657
PRJEB2214	ERS177717	ERX151707	ERR175561	A_MH2145	5965_2_2	2145
PRJEB2214	ERS177718	ERX151708	ERR175562	A_MH2497	5965_2_3	2497
PRJEB2214	ERS177719	ERX151709	ERR175563	A_MH5368	5965_2_4	5368
PRJEB2214	ERS177720	ERX151710	ERR175564	A_MH5786	5965_2_5	5786
PRJEB2214	ERS177721	ERX151711	ERR175565	A_MH12023	5965_2_6	12023
PRJEB2214	ERS177722	ERX151712	ERR175566	A_MH13849	5965_2_7	13849
PRJEB2214	ERS177723	ERX151713	ERR175567	A_MH14553	5965_2_8	14553
PRJEB2214	ERS177724	ERX151714	ERR175568	A_MH16005	5965_2_9	16005
PRJEB2214	ERS177727	ERX151717	ERR175571	A_MH23529	5965_3_1	23529
PRJEB2214	ERS177736	ERX151726	ERR175580	A_MH8910	5965_3_10	8910
PRJEB2214	ERS177737	ERX151727	ERR175581	A_MH9922	5965_3_11	9922
PRJEB2214	ERS177728	ERX151718	ERR175572	A_MH24673	5965_3_2	24673
PRJEB2214	ERS177729	ERX151719	ERR175573	A_MH25256	5965_3_3	25256
PRJEB2214	ERS177730	ERX151720	ERR175574	A_MH47300	5965_3_4	47300
PRJEB2214	ERS177731	ERX151721	ERR175575	A_MH1364	5965_3_5	1364
PRJEB2214	ERS177732	ERX151722	ERR175576	A_MH3234	5965_3_6	3234
PRJEB2214	ERS177733	ERX151723	ERR175577	A_MH4510	5965_3_7	4510
PRJEB2214	ERS177734	ERX151724	ERR175578	A_MH6446	5965_3_8	6446
PRJEB2214	ERS177735	ERX151725	ERR175579	A_MH7205	5965_3_9	7205
PRJEB2214	ERS177738	ERX151728	ERR175582	A_MH10549	5965_4_1	10549
PRJEB2214	ERS177747	ERX151737	ERR175591	A_MH17127	5965_4_10	17127
PRJEB2214	ERS177748	ERX151738	ERR175592	A_MH18876	5965_4_11	18876
PRJEB2214	ERS177739	ERX151729	ERR175583	A_MH10648	5965_4_2	10648

PRJEB2214	ERS177740	ERX151730	ERR175584	A_MH10901	5965_4_3	10901
PRJEB2214	ERS177741	ERX151731	ERR175585	A_MH11715	5965_4_4	11715
PRJEB2214	ERS177742	ERX151732	ERR175586	A_MH11979	5965_4_5	11979
PRJEB2214	ERS177743	ERX151733	ERR175587	A_MH15048	5965_4_6	15048
PRJEB2214	ERS177744	ERX151734	ERR175588	A_MH15741	5965_4_7	15741
PRJEB2214	ERS177745	ERX151735	ERR175589	A_MH16170	5965_4_8	16170
PRJEB2214	ERS177746	ERX151736	ERR175590	A_MH16665	5965_4_9	16665
PRJEB2214	ERS177749	ERX151739	ERR175593	A_MH19679	5965_5_1	19679
PRJEB2214	ERS177758	ERX151748	ERR175602	A_MH34496	5965_5_10	34496
PRJEB2214	ERS177750	ERX151740	ERR175594	A_MH20130	5965_5_2	20130
PRJEB2214	ERS177751	ERX151741	ERR175595	A_MH20933	5965_5_3	20933
PRJEB2214	ERS177752	ERX151742	ERR175596	A_MH21571	5965_5_4	21571
PRJEB2214	ERS177753	ERX151743	ERR175597	A_MH24519	5965_5_5	24519
PRJEB2214	ERS177754	ERX151744	ERR175598	A_MH24640	5965_5_6	24640
PRJEB2214	ERS177755	ERX151745	ERR175599	A_MH25883	5965_5_7	25883
PRJEB2214	ERS177756	ERX151746	ERR175600	A_MH26862	5965_5_8	26862
PRJEB2214	ERS177757	ERX151747	ERR175601	A_MH27137	5965_5_9	27137
PRJEB2214	ERS177759	ERX151749	ERR175603	A_MH35739	5965_6_1	35739
PRJEB2214	ERS177760	ERX151750	ERR175604	A_MH53658	5965_6_2	53658
