# Supplementary material for: Population-based analysis of ocular Chlamydia trachomatis in trachoma-endemic West African communities identifies genomic markers of disease severity
Source: Genome Med. 2018 Feb 26;10:15. doi: 10.1186/s13073-018-0521-x (PMC5828069; doi:10.1186/s13073-018-0521-x)
Supplement: Supplementary file 5 — Figure S5. Maximum likelihood reconstruction of the ompA (CTA0742) phylogeny. (PDF 450 kb) [file 13073_2018_521_MOESM5_ESM.pdf]

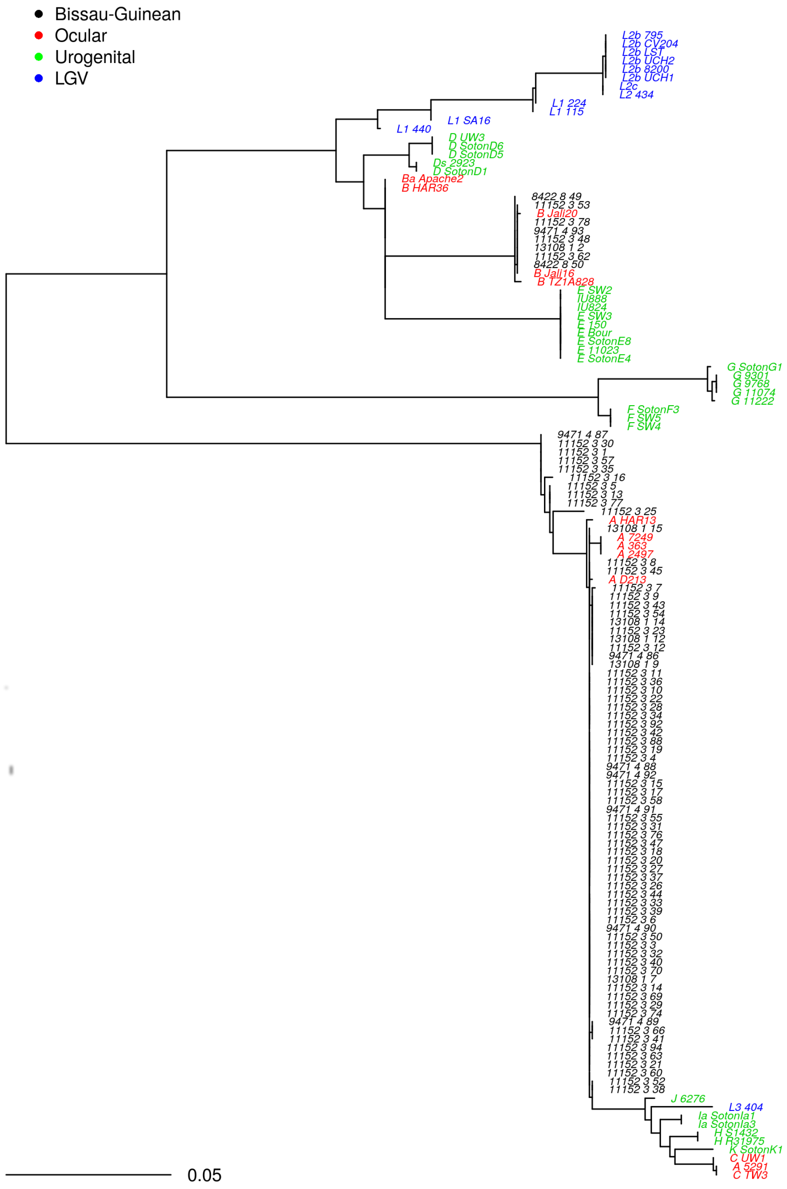

Figure S5. Maximum likelihood reconstruction of the ompA (CTA0742) phylogeny. Maximum likelihood reconstruction of the whole-genome phylogeny of 81 *Ct* sequences from the Bijagós Islands and 54 *Ct* reference strains. Bijagós *Ct* sequences (n=81) were mapped to Ct A/HAR-13 using SAMtools [55]. SNPs were called as described by Harris *et al.* [4]. Phylogenies were computed with RAxML [62] from a variable sites alignment using a GTR+gamma model and are midpoint rooted. The scale bar indicates evolutionary distance. Bijagós *Ct* sequences in this study are coloured BLACK and reference strains are coloured by tissue localization (RED=Ocular, GREEN=Urogenital, BLUE=LGV). Branches are supported by > 90% of 1000 bootstrap replicates. Branches supported by 80-90% (ORANGE) and < 80% (BROWN) bootstrap replicates are indicated.
